# Dynamic postnatal development of the cellular and circuit properties of striatal D1 and D2 spiny projection neurons

**DOI:** 10.1113/JP278416

**Published:** 2019-10-10

**Authors:** Rohan N. Krajeski, Anežka Macey‐Dare, Fran van Heusden, Farid Ebrahimjee, Tommas J. Ellender

**Affiliations:** ^1^ Department of Pharmacology University of Oxford Oxford OX1 3QT UK

## Abstract

**Key points:**

- Imbalances in the activity of the D1‐expressing direct pathway and D2‐expressing indirect pathway striatal projection neurons (SPNs) are thought to contribute to many basal ganglia disorders, including early‐onset neurodevelopmental disorders such as obsessive–compulsive disorder, attention deficit hyperactivity disorder and Tourette's syndrome.
- This study provides the first detailed quantitative investigation of development of D1 and D2 SPNs, including their cellular properties and connectivity within neural circuits, during the first postnatal weeks.
- This period is highly dynamic with many properties changing, but it is possible to make three main observations: many aspects of D1 and D2 SPNs progressively mature in parallel; there are notable exceptions when they diverge; and many of the defining properties of mature striatal SPNs and circuits are already established by the first and second postnatal weeks, suggesting guidance through intrinsic developmental programmes.
- These findings provide an experimental framework for future studies of striatal development in both health and disease.

**Abstract:**

Many basal ganglia neurodevelopmental disorders are thought to result from imbalances in the activity of the D1‐expressing direct pathway and D2‐expressing indirect pathway striatal projection neurons (SPNs). Insight into these disorders is reliant on our understanding of normal D1 and D2 SPN development. Here we provide the first detailed study and quantification of the striatal cellular and circuit changes occurring for both D1 and D2 SPNs in the first postnatal weeks using *in vitro* whole‐cell patch‐clamp electrophysiology. Characterization of their intrinsic electrophysiological and morphological properties, the excitatory long‐range inputs coming from cortex and thalamus, as well their local gap junction and inhibitory synaptic connections reveals this period to be highly dynamic with numerous properties changing. However it is possible to make three main observations. Firstly, many aspects of SPNs mature in parallel, including intrinsic membrane properties, increases in dendritic arbours and spine densities, general synaptic inputs and expression of specific glutamate receptors. Secondly, there are notable exceptions, including a transient stronger thalamic innervation of D2 SPNs and stronger cortical NMDA receptor‐mediated inputs to D1 SPNs, both in the second postnatal week. Thirdly, many of the defining properties of mature D1 and D2 SPNs and striatal circuits are already established by the first and second postnatal weeks, including different electrophysiological properties as well as biased local inhibitory connections between SPNs, suggesting this is guided through intrinsic developmental programmes. Together these findings provide an experimental framework for future studies of D1 and D2 SPN development in health and disease.

## Key points

- Imbalances in the activity of the D1‐expressing direct pathway and D2‐expressing indirect pathway striatal projection neurons (SPNs) are thought to contribute to many basal ganglia disorders, including early‐onset neurodevelopmental disorders such as obsessive–compulsive disorder, attention deficit hyperactivity disorder and Tourette's syndrome.
- This study provides the first detailed quantitative investigation of development of D1 and D2 SPNs, including their cellular properties and connectivity within neural circuits, during the first postnatal weeks.
- This period is highly dynamic with many properties changing, but it is possible to make three main observations: many aspects of D1 and D2 SPNs progressively mature in parallel; there are notable exceptions when they diverge; and many of the defining properties of mature striatal SPNs and circuits are already established by the first and second postnatal weeks, suggesting guidance through intrinsic developmental programmes.
- These findings provide an experimental framework for future studies of striatal development in both health and disease.

## Introduction

The striatum is the main input nucleus of the basal ganglia and consists of two populations of projection neurons with distinct long‐range outputs, the D1‐expressing direct pathway spiny projection neurons (SPNs) and the D2‐expressing indirect pathway SPNs (Day *et al*. 2008; Gertler *et al*. 2008), which differentially regulate motor behaviour and cognitive function (Graybiel *et al*. 1994; Grillner *et al*. 2005; Yin & Knowlton, 2006; Kravitz *et al*. 2010; Tecuapetla *et al*. 2016). Adult D1 and D2 SPNs exhibit distinct electrical and morphological properties (Gertler *et al*. 2008) and form precise non‐random local synaptic connections with each other (Taverna *et al*. 2008; Planert *et al*. 2010; Cepeda *et al*. 2013). Imbalance in the activity of the two pathways is thought to contribute to the cognitive and motor symptoms seen in late onset neurodegenerative disorders such as Parkinson's disease (Taverna *et al*. 2008) and Huntington's disease (Cepeda *et al*. 2013), but also those seen in early onset neurodevelopmental disorders such as Tourette's syndrome (McNaught & Mink, 2011; Albin, 2018), obsessive–compulsive disorder (Graybiel & Rauch, 2000; Langen *et al*. 2011), attention deficit hyperactivity disorder (Del Campo *et al*. 2011) and autism spectrum disorders (Shepherd, 2013). The cellular and neural circuit changes that underpin these neurodevelopmental disorders are major research areas. Although key papers have started to shed light on early postnatal striatal development (Tepper *et al*. 1998; Dehorter *et al*. 2011; Kozorovitskiy *et al*. 2012; Peixoto *et al*. 2016), often SPNs have been grouped together as one population and therefore many aspects of D1 and D2 SPN postnatal development remain unknown.

A combination of whole‐cell patch‐clamp electrophysiology and anatomical analysis in mouse brain slices allows for the investigation of the cellular and circuit properties of striatal D1 and D2 SPNs from the earliest postnatal periods into maturity. These include postnatal day (P)3–6, the period when most striatal SPNs have been born but excitatory synaptic input to the striatum is thought to be minimal and mouse pups produce little movement; P9–12, when excitatory synaptic inputs to the striatum are thought to have undergone a period of rapid maturation and motor competence of the pups has increased; P21–28, when the striatal neurons and the circuit are approaching maturity and mice readily traverse the environment; and finally P35+, when the brain is thought to have reached maturity coinciding with the sexual maturity of mice (Finlay & Darlington, 1995; Tepper *et al*. 1998; Khazipov *et al*. 2004; Dehorter *et al*. 2011; Kozorovitskiy *et al*. 2012; Peixoto *et al*. 2016). Overall, we found that the early postnatal development of striatal D1 and D2 SPNs is highly dynamic with many intrinsic and circuit properties changing. We found that young D1 SPNs are electrophysiologically more mature than D2 SPNs and that intrinsic electrophysiological differences between adult D1 and D2 SPNs are already apparent in the second postnatal week. Both D1 and D2 SPNs exhibit similar increases in dendritic arbour and spine density and equally sample excitatory cortical and thalamic inputs in the first postnatal week. Subsequent maturation of excitatory synapses occurs mostly in parallel and is relatively rapid for thalamic synapses and more prolonged for cortical synapses. The notable exception is a transient strong input to D2 SPNs from thalamus and a stronger NMDA receptor‐mediated input to D1 SPNs, both in the second postnatal week. All excitatory inputs in the second postnatal week are further characterized by their long durations and decay times and pharmacological study suggests this is mediated through expression of specific combinations of glutamate receptors. Inhibitory synapses onto SPNs are initially sparser and exhibit a more prolonged maturation, as reflected by a progressive increase in miniature inhibitory postsynaptic current (mIPSC) frequency. Indeed, simultaneous quadruple patch‐clamp recordings and the study of local connections between developing SPNs reveals that in the first postnatal week SPNs mainly form gap junctions with each other which only in later postnatal weeks are increasingly replaced by inhibitory synaptic connections. Interestingly, these early inhibitory synaptic connections are precise and non‐random and relative biases in synaptic connectivity found in adulthood are already apparent in the second postnatal week, including highly interconnected D2 SPNs. Together, these results suggest that striatal D1 and D2 SPN postnatal development is both highly dynamic and organized with many of the cellular and circuit properties established soon after birth suggesting a role for intrinsic developmental programmes in guiding their early development.

## Methods

### Ethical approval

The present study conforms to the ethical principles and regulations of *The Journal of Physiology* and with *The Journal*’s animal ethics checklist as described by Grundy (2015). All animal work performed at the University of Oxford (UK) was licensed by the Home Office under the Animals (Scientific Procedures) Act 1986 and was approved by the University of Oxford Ethical Review Committee. All efforts were taken to minimize animal numbers.

### Animals

All experiments were carried out on C57/BL6 wild‐type and heterozygous D1‐GFP or D2‐GFP mice of both sexes with *ad libitum* access to food and water. The D1‐GFP or D2‐GFP bacterial artificial chromosome (BAC) transgenic mice report subtypes of the dopamine receptor, either D1 or D2, by the presence of green fluorescent protein (GFP) (Mutant Mouse Regional Resource Centres (MMRRC), USA). Details of the mice and the methods of BAC mice production have been published (Gong *et al*. 2003) and can be found on the GENSAT website (GENSAT (2009) The Gene Expression Nervous System Atlas (GENSAT) Project. In: NINDS, Contracts N01NS02331 and HHSN271200723701C, The Rockefeller University (New York, USA), http://www.gensat.org/index.html). In brief, the genotype of the mice has been modified to contain multiple copies of a modified BAC in which the enhanced GFP (EGFP) reporter gene is inserted immediately upstream of the coding sequence of the D1 or D2 gene. These BAC transgenic mice arrived originally on a Swiss Webster background but were backcrossed to a C57/BL6 background over 20+ generations prior to use and kept as a heterozygous mouse line to avoid published issues using these transgenic lines (Bagetta *et al*. 2011; Kramer *et al*. 2011; Chan *et al*. 2012; Nelson *et al*. 2012). Experiments were designed to use litter mates for the various age ranges within single experiments so as to control for effects of litter sizes and maternal care factors that could affect levels of neuronal and circuit maturity. All mice were bred, individually ventilated cage (IVC) housed in a temperature controlled animal facility (normal 12:12 h light–dark cycles) and used in accordance with the UK Animals (Scientific Procedures) Act (1986).

### Slice preparation and recording conditions

Acute striatal slices were made from postnatal animals at postnatal day (P)3–6, P9–12, P21–28 and P35 and older. Animals were anaesthetized with isoflurane and then decapitated. Coronal and horizontal (for thalamic experiments only) 350–400 µm slices were cut using a vibrating‐blade microtome (Microm HM650V, Walldorf, Germany). Slices were prepared in artificial cerebrospinal fluid (aCSF) containing (in mm): 65 sucrose, 85 NaCl, 2.5 KCl, 1.25 NaH_2_PO_4_, 7 MgCl_2_, 0.5 CaCl_2_, 25 NaHCO_3_ and 10 glucose, pH 7.2–7.4, bubbled with carbogen gas (95% O_2_–5% CO_2_). Slices were immediately transferred to a storage chamber containing aCSF (in mm): 130 NaCl, 3.5 KCl, 1.2 NaH_2_PO_4_, 2 MgCl_2_, 2 CaCl_2_, 24 NaHCO_3_ and 10 glucose, pH 7.2–7.4, at 32°C and bubbled with carbogen gas until used for recording. Striatal slices were transferred to a recording chamber and continuously superfused with aCSF bubbled with carbogen gas with the same composition as the storage solution (32°C and perfusion speed of 2 ml min^−1^). Whole‐cell current‐clamp recordings were performed using glass pipettes (∼6 MΩ), pulled from standard wall borosilicate glass capillaries and containing for whole‐cell current‐clamp (in mm): 110 potassium gluconate, 40 Hepes, 2 ATP‐Mg, 0.3 Na‐GTP, 4 NaCl and 4 mg ml^−1^ biocytin (pH 7.2–7.3; osmolarity, 290–300 mosmol l^−1^) and for whole‐cell voltage‐clamp (in mm): 120 caesium gluconate, 40 Hepes, 4 NaCl, 2 ATP‐Mg, 0.3 Na‐GTP, 0.2 QX‐314 and 4 mg ml^−1^ biocytin (pH 7.2–7.3; osmolarity, 290–300 mosmol l^−1^). For recordings of putative gap junctions and synaptic connections between SPNs, an intracellular solution with high internal [Cl^−^] was used containing (in mm): 105 potassium gluconate, 30 KCl, 10 Hepes, 4 ATP‐Mg, 0.3 Na‐GTP and 4 mg ml^−1^ biocytin (pH 7.2–7.3; osmolarity, 290–300 mosmol l^−1^). Liquid junction potentials were not corrected for. Succesfully patched neurons in all age ranges could be maintained and were healthy for at least 30 min although neurons in the P3–6 and P9–12 age groups tended to deteriorate faster on average than those in the older groups. Recordings were made using Multiclamp 700B amplifiers (Molecular Devices, Wokingham, UK) and filtered at 4 kHz and acquired using an InstruTECH ITC‐18 analog/digital board (Digitimer, Welwyn Garden City, UK) and WinWCP software (University of Strathclyde, UK, RRID:SCR_014713) at 10 kHz.

### Stimulation and recording protocols

Hyperpolarizing and depolarizing current steps were used to assess the intrinsic properties of the recorded SPNs including input resistance, spike threshold (using small incremental current steps) and membrane time constant, as well as the properties of action potentials (amplitude, frequency and duration). Properties were assessed immediately on break‐in. Currents step ranges were for P3–6: −50 to +50 pA; for P9–12: −100 to +100 pA; and for P21–28 and P35+: −500 to +500 pA. These ranges of currents were chosen to allow sufficient depolarization of SPNs taking into consideration changes in input resistance and observations of depolarization block and action potential failure in SPNs. In a subset of neurons the membrane capacitance was assessed from the area under the capacitive transient as a result of repeated voltage steps (‘seal test’, 5 mV, at 20 Hz) in voltage‐clamp mode. Both miniature excitatory postsynaptic currents (mEPSCs) and mIPSCs were recorded from individual SPNs, held at respectively −75 and 0 mV in aCSF containing 1 µm TTX, in 5 min sweeps. Neurons were kept for 5+ minutes in whole‐cell configuration mode before miniature recordings started to facilitate ionic and QX‐314 diffusion of the intracellular solution. Activation of excitatory cortical and thalamic afferents was performed via a bipolar stimulating electrode (FHC Inc., Bowdoin, ME, USA) placed in respectively the external or internal capsule, and in the presence of blockers of inhibitory GABAergic transmission including the GABA_A_ receptor antagonist SR95531 (1 µm) and the GABA_B_ receptor antagonist CGP52432 (2 µm). Afferents were activated every 5 s with up to 20 repetitions and excitatory postsynaptic currents (EPSCs) and excitatory postsynaptic potentials (EPSPs) were recorded from the patched SPNs. Evoked EPSCs were recorded in whole‐cell voltage‐clamp mode at a holding potential near −75 mV and evoked EPSPs in whole‐cell current‐clamp mode at resting membrane potential. Trains of stimulations consisted of 10 pulses given at 20 Hz and trains were repeated every 30 s up to 5 times. Combined AMPA/kainate and NMDA receptor‐mediated currents were recorded from SPNs held at +50 mV. AMPA/kainate receptor‐mediated currents were recorded after a 5–10 min wash‐in of the NMDA receptor antagonist d‐(−)‐2‐amino‐5‐phosphonopentanoic acid (d‐ap5 ; 50 µm). The contribution of different glutamate receptor subtypes to striatal evoked EPSPs was investigated using superfusion of the NR2C/D subunit‐selective NMDA receptor antagonist 2*S* ^*^,3*R* ^*^‐1‐(Phenanthren‐2‐carbonyl)piperazine‐2,3‐dicarboxylic acid (PPDA) (200 nm), the NMDA receptor antagonist d‐AP5 (50 µm), the AMPA/kainate receptor antagonist 2,3‐dihydroxy‐6‐nitro‐7‐sulfamoyl‐benzo[f]quinoxaline‐2,3‐dione (NBQX; 20 µm) and the kainate receptor antagonist UBP‐310 (5 µm). All drugs were obtained from Tocris Biosciences (Bristol, UK). Local gap junctions between SPNs were examined by delivering hyperpolarizing current injections (200 ms, P3–6: −20 pA; P9–12: −100 pA; and P21–28: −200 pA) to each patched SPN sequentially, whilst simultaneously monitoring the membrane voltage of the other SPNs. Local inhibitory synaptic connectivity between SPNs was examined by delivering brief (∼60 ms) suprathreshold current injections (P3–6: +50 pA; P9–12: +150 pA; and P21–28: +400 pA) or brief trains of current injections (6 pulses, 30 ms, P3–6: +80 pA; P9–12: +200 pA; and P21–28: +500 pA at 20 Hz) to each patched SPN sequentially, whilst simultaneously monitoring the membrane voltage of the other SPNs. Protocols were repeated 20–30 times for the detection of gap junctions and synaptic connections.

### Analysis of recordings

Data were analysed offline using custom written programmes in Igor Pro (Wavemetrics, Lake Oswego, OR, USA, RRID:SCR_000325). The input resistance was calculated from the observed membrane potential change after hyperpolarizing the membrane potential with a set current injection. The membrane time constant was taken as the time it takes for a change in potential to reach 63% of its final value. The spike threshold was the membrane voltage at which the SPN generated an action potential. The action potential amplitude was taken from the peak amplitude of the individual action potentials relative to the average steady‐state membrane depolarization during positive current injection. Action potential duration was taken as the duration between the upward and downward stroke of the action potential at 25% of the peak amplitude. mEPSCs and mIPSCs were detected as downward and upward deflections of more than 2 standard deviations (SD) above baseline (baseline consisted of the average holding current across the entire recording) and more than 10 ms duration in 5 min duration traces which were lowpass filtered at 50 Hz. Miniature events were not corrected for developmental changes in membrane capacitance. Evoked EPSCs, EPSPs and inhibitory postsynaptic potentials (IPSPs) were defined as upward or downward deflections of more than 2 SD on average synaptic responses generated after filtering and averaging original traces (0.1 Hz high‐pass filter and 500 Hz low‐pass filter) and used for analysis of synaptic properties. Synaptic properties include measurements of peak amplitude, duration (measured from the start of the upward/downward stroke of the event until its return to the pre‐event baseline), rise time (time between 20% and 80% of the peak amplitude) and decay time (measured as the time from peak amplitude until the event returned to 50% of peak amplitude). Synaptic delays were calculated from the time of stimulation to the start of the upward stroke of the synaptic response. The short‐term plastic properties of cortical and thalamic excitatory synapses and inhibitory synapses between SPNs were analysed by taking the amplitude of each EPSP/IPSP during train stimulation and dividing this by the amplitude of the first response. The NMDA/AMPA ratio was calculated from recordings of the combined AMPA/kainate and NMDA receptor‐mediated current as well as the pharmacologically isolated AMPA/kainate receptor‐mediated current. The average AMPA/kainate receptor‐mediated current trace was subtracted from the combined AMPA/kainate and NMDA receptor‐mediated current trace to obtain the NMDA receptor‐mediated current. Peak amplitude NMDA receptor‐mediated current was divided by peak amplitude AMPA/kainate receptor‐mediated current to obtain the NMDA/AMPA ratio. The presence of gap junctions was assessed by averaging the 20–30 sweeps consisting of hyperpolarizing current injections and observing a significant downward deflection of more than 2 SD from baseline. The coupling coefficient (CC) was obtained by dividing the amplitude of the low‐frequency voltage change in the receiver SPN to that in the driver SPN. The junctional conductance (*G*_j_) was estimated from *R*_input_ and CC (Venance *et al*. 2004): *G*_j1–2_ = *R*_input1_ × CC_1–2_/[(*R*_input1_ × *R*_input2_) − (*R*_input1_ × CC_1–2_)^2^], where *R*_input1_ and *R*_input2_ are the *R*_input_ values of the injected and receiving SPNs, respectively, and CC_1–2_ the CC between the injected and receiving SPNs.

### Histological analyses and cell classification

Following whole‐cell patch‐clamp recordings, the brain slices were fixed in 4% paraformaldehyde in 0.1 m phosphate buffer (PB; pH 7.4). Biocytin‐filled neurons were visualized by incubating sections in 1:10,000 streptavidin AlexaFluor405‐conjugated antibodies (Thermo Fisher Scientific, Waltham, MA, USA, cat. no. S32351). Visualized neurons were labelled for chicken ovalbumin upstream promoter transcription‐factor interacting protein‐2 (CTIP2; 1:1000, rat, Abcam (Cambridge, UK), cat. no. ab14865, RRID:AB_2064130) and pre‐proenkephalin (PPE; 1:1000, rabbit, LifeSpan Biosciences (Seattle, WA, USA), cat. no. LS‐C23084, RRID:AB_902714) in PBS containing 0.3% Triton X‐100 (PBS‐Tx) overnight at 4 °C followed by incubation with goat anti‐rat AlexaFluor647 (1:500; Thermo Fisher Scientific, cat. no. A‐21247, RRID:AB_141778) and goat anti‐rabbit AlexaFluor555 (1:500; Thermo Fisher Scientific, cat. no. A‐21429, RRID:AB_2535850) secondary antibodies in 0.3% PBS‐Tx for 2 h at room temperature for D1 or D2 SPN classification. CTIP2 is expressed by SPNs and not interneurons (Arlotta *et al*. 2008) and PPE reliably labels indirect pathway D2 SPNs (Lee *et al*. 1997; Sharott *et al*. 2017). PPE antibody staining was facilitated through antigen retrieval by heating sections at 80°C in 10 mm sodium citrate (pH 6.0) for approximately 30–60 min prior to incubation with PPE primary antibody. After classification of SPNs the slices were washed 3 times in PBS and processed for 3,3′‐diaminobenzidine (DAB) immunohistochemistry using standard procedures. Fluorescence images were captured with a LSM 710 confocal microscope using ZEN software (Zeiss, Cambridge, UK; RRID:SCR_013672). DAB‐immunoreactive neurons were visualized on a brightfield microscope and were reconstructed and analysed using Neurolucida and Neuroexplorer software (MBF Bioscience, Delft, The Netherlands; RRID:SCR_001775). Only labelled neurons that exhibited a full dendritic arbour were included for analysis, e.g. cells with clear truncations were not included in the dataset. Scholl analysis and polarity analysis were performed using standard procedures. In brief, both Scholl and polarity plots were generated for individual SPNs by calculating the total dendritic length located within 10° segments with increasing distance from the soma. The dendritic lengths were subsequently normalized for an individual SPN and averaging the normalized plots of individual neurons generated final plots.

### Statistics

**Figure 1:**
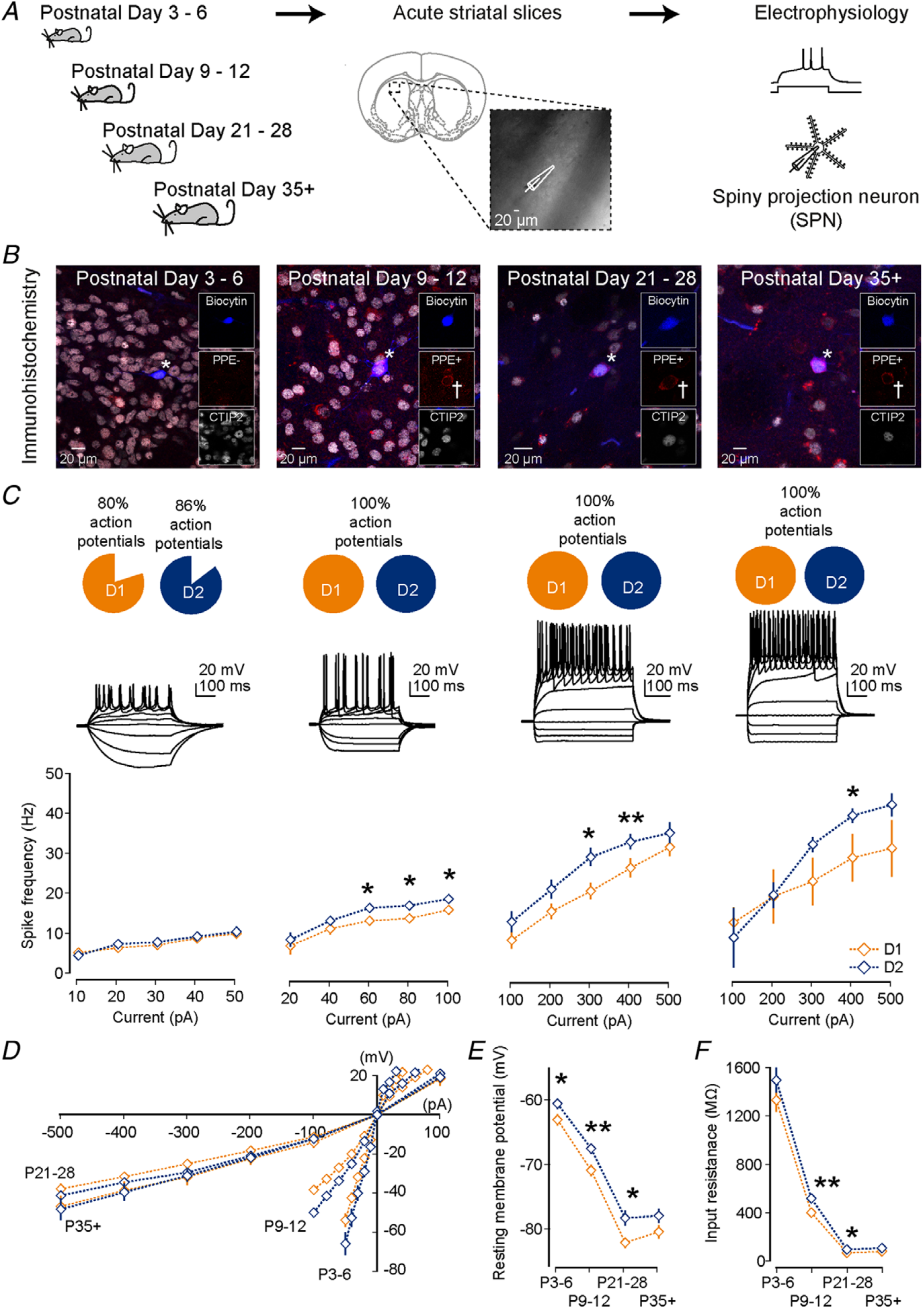
Maturational and intrinsic differences in electrophysiological properties of striatal D1 and D2 SPNs *A*, whole‐cell patch‐clamp recordings were made from striatal spiny projection neurons (SPNs) in acute coronal brain slices of mice at four developmental stages: postnatal day (P)3–6, P9–12, P21–28 and P35 and older. *B*, internal recording solutions included biocytin allowing for *post hoc* confirmation of SPN type using immunocytochemistry for the SPN marker CTIP2 and the D2 SPN marker PPE. Recorded SPNs are indicated by asterisks. Note the example SPN at P3–6 is PPE‐negative and CTIP2‐positive, corresponding to a putative D1 SPN, whereas the SPNs for other age ranges are PPE‐positive (as indicated by crosses) and CTIP2‐positive, corresponding to putative D2 SPNs. *C*, hyperpolarizing and depolarizing current steps were used to characterize the electrophysiological properties of SPNs. We found that the majority of SPNs were already able to generate small action potentials from P3–6 onwards. Whereas in the first postnatal week both D1 and D2 SPNs exhibit similar action potential frequencies, at later stages in development D2 SPNs start exhibiting a significantly higher action potential frequency persisting into adulthood. *D*, a pronounced inward rectifying current develops as both D1 and D2 SPNs mature. *E*, the resting membrane potential becomes progressively more hyperpolarized as SPNs mature. Note the consistently more depolarized resting membrane potential of the D2 SPNs. *F*, the input resistance progressively decreases as SPNs mature. Note the consistently higher input resistance of the D2 SPNs.

**Figure 2:**
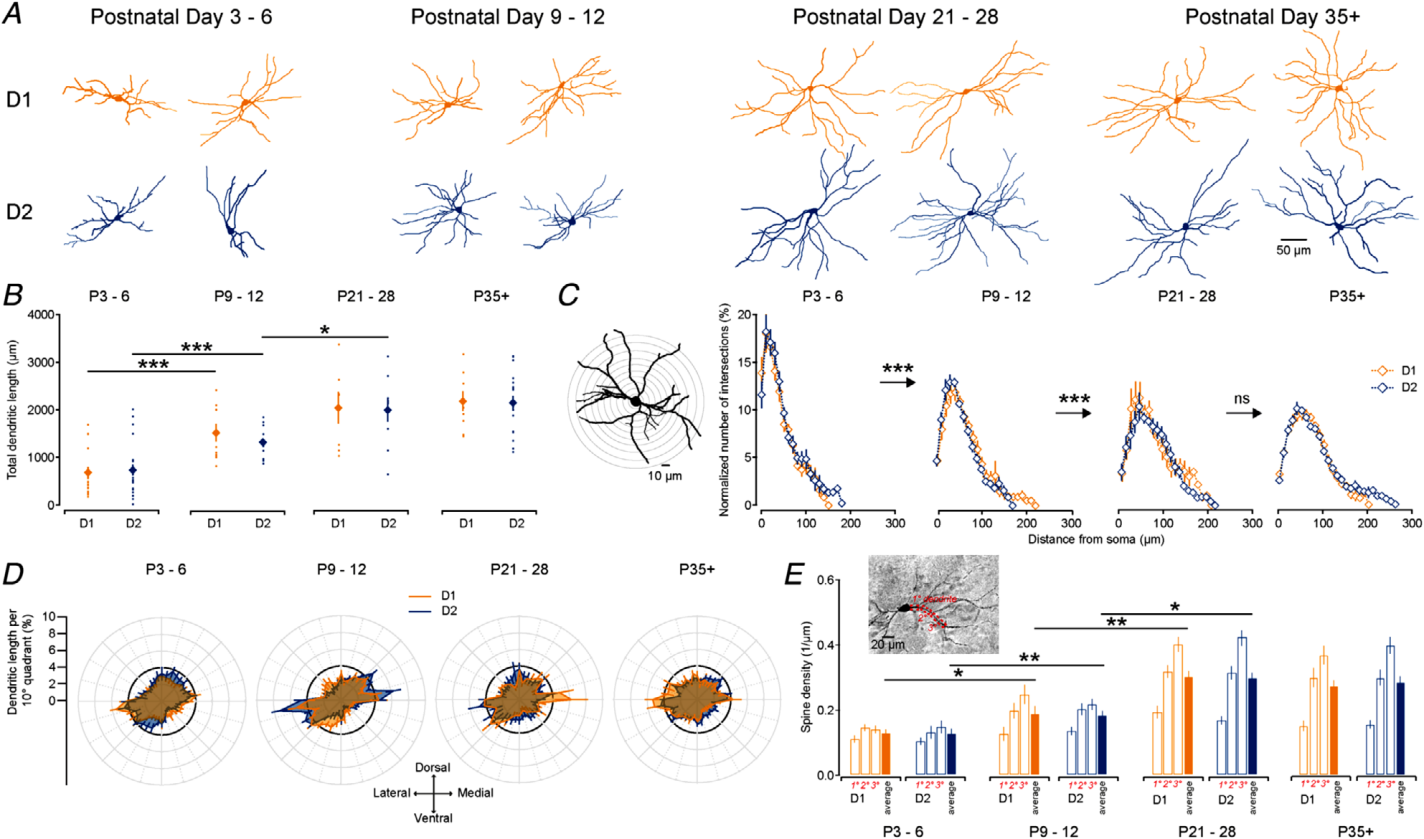
Development of dendritic arbours and spines of D1 and D2 SPNs *A*, example reconstructions of previously recorded SPNs processed for DAB immunohistochemistry. SPNs are grouped according to age (left to right, P3–6, P9–12, P21–28 and P35+) and whether they are D1 (orange, top) or D2 (blue, bottom) SPNs. The examples shown are all reconstructed and analysed neurons from coronal sections and are aligned such that top is dorsal, bottom is ventral, left is lateral and right is medial. *B*, D1 (orange) and D2 (blue) SPNs exhibit a significant and similar increase in their dendritic length as they mature *C*, Scholl analysis of dendritic complexity of D1 and D2 SPNs reveals a similar elaboration of distal dendritic segments as they mature. *D*, polarity analysis of dendrites of D1 and D2 SPNs reveals a mostly uniform and radial distribution of their dendrites. Note the bias to extend dendrites from lateral–ventral aspects to medial–dorsal aspects. *E*, spine density of D1 and D2 SPNs in different age ranges. Note the similar increase in spine density in both D1 and D2 SPNs as they mature.

**Figure 3:**
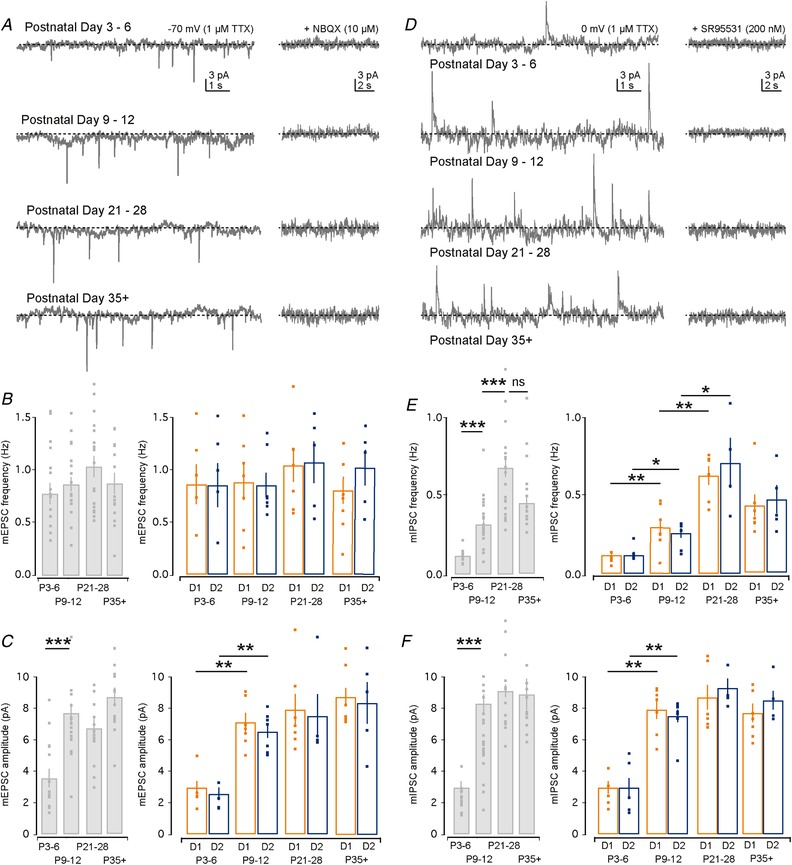
Characterization of mEPSCs and mIPSCs in D1 and D2 SPNs *A*, mEPSCs were recorded as downward deflections from SPNs held in voltage‐clamp at a holding potential of −70 mV in the presence of TTX (1 µm). *B*, bar plot of mEPSC frequency showing a relatively stable mEPSC frequency across development for all recorded SPNs (left) and identified D1 (orange) and D2 (blue) SPNs (right). Note the absence of significant differences in mEPSC frequency between D1 and D2 SPNs. *C*, bar plot of mEPSC amplitude for all recorded SPNs showing a significant increase in amplitude between P3–6 and P9–12 (*P* < 0.0001, left). This increase in amplitude is seen for both the D1 and D2 SPNs (right). *D*, mIPSCs were recorded as upward deflections from SPNs held in voltage‐clamp at a holding potential of 0 mV in the presence of TTX (1 µm). *E*, bar plot of mIPSC frequency showing a steady increase across the early developmental age ranges (P3–6 to P9–12, *P* < 0.0001, and P9–12 to P21–28, *P* < 0.0001, left), which is seen for both D1 and D2 SPNs (right), after which there is a slight, but insignificant (*P* = 0.526), drop in frequency. *F*, bar plot of mIPSC amplitude showing a significant increase between P3–6 and P9–12 (*P* < 0.0001, left) for both D1 and D2 SPNs (right).

**Figure 4:**
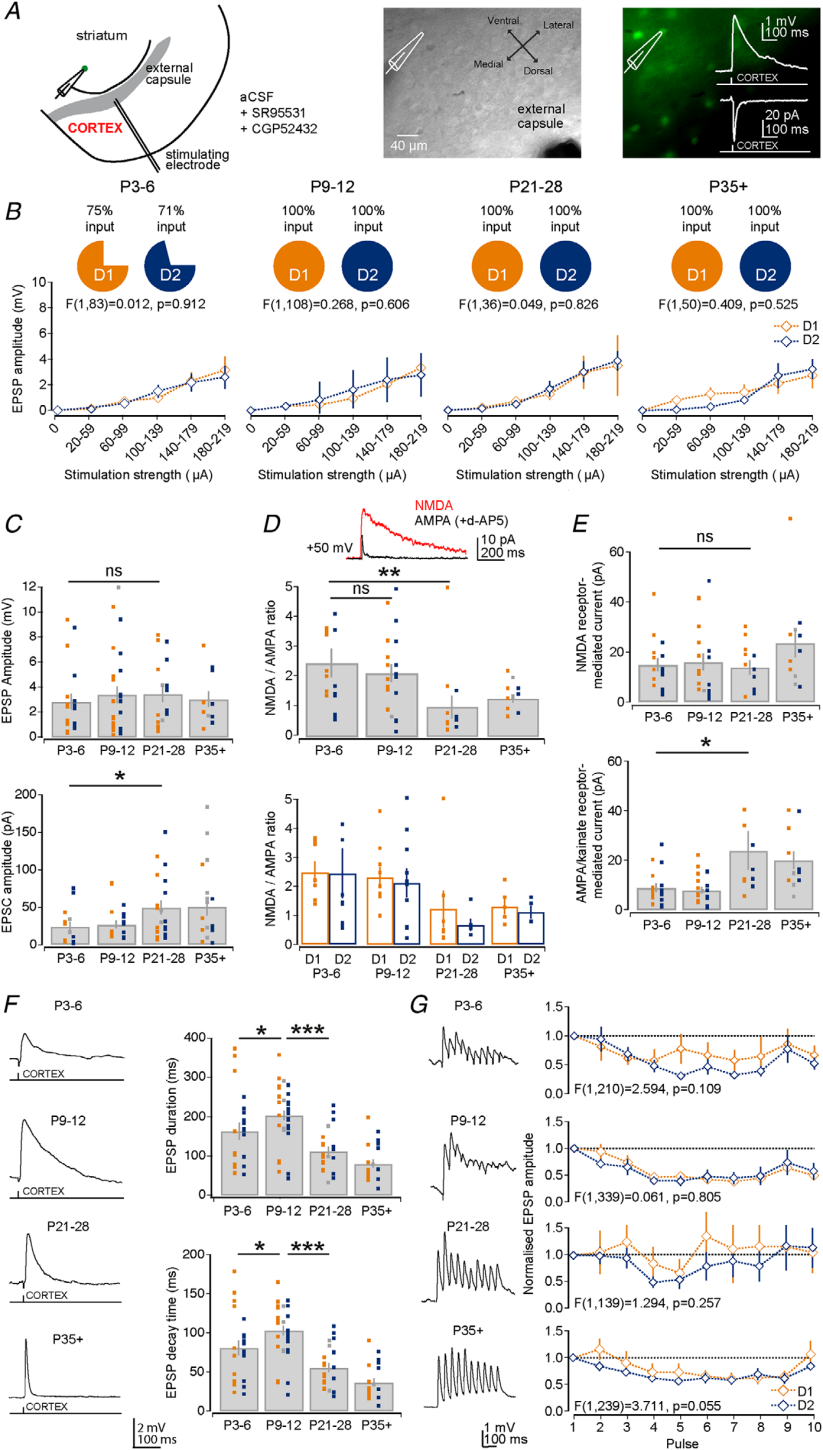
Development of cortical excitatory synaptic inputs onto D1 and D2 SPNs *A*, diagram of the recording configuration and placement of stimulation electrode to activate cortical afferents (left). Example Dodt contrast image (middle) and fluorescence image (right) of the striatum in a D2–GFP transgenic mouse. Inset: example of an evoked cortical EPSP and EPSC. *B*, graphs of EPSP amplitude across a range of stimulation strengths (range 20–220 µA) for the four different age ranges. Note the similar amplitude in evoked EPSPs between the D1 (orange) and D2 (blue) SPNs. Also, note that ∼70% of SPNs exhibited a response at P3–6 and responses could always be observed at later ages. *C*, bar plot of the maximum evoked EPSP amplitude across the age ranges which remains relatively constant at ∼3 to 4 mV (top). Bar plot of EPSC amplitude shows an increase in the cortically evoked excitatory current (bottom), especially evident from P9–12 to P21–28. *D*, bar plots of the NMDA/AMPA ratio across the age ranges. Note the decrease in the ratio as the neurons mature (top), which occurs in parallel for both D1 and D2 SPNs (bottom). *E*, bar plots of the NMDA receptor‐mediated current (top) and AMPA/kainate receptor‐mediated current (bottom) across the age ranges. Note the significant increase in the AMPA/kainate receptor‐mediated current whereas the NMDA receptor‐mediated current stayed constant. *F*, bar plots of the EPSP duration and decay time. Note the transient and significant increase in the EPSP duration and decay time at P9–12. *G*, graphs of the EPSP amplitude across 10 stimulations at 20 Hz showing that corticostriatal synapses at D1 and D2 SPNs predominantly exhibit short‐term depression at all age ranges. D1 SPNs: orange squares; D2 SPNs: blue squares; and unclassified SPNs: grey squares.

**Figure 5:**
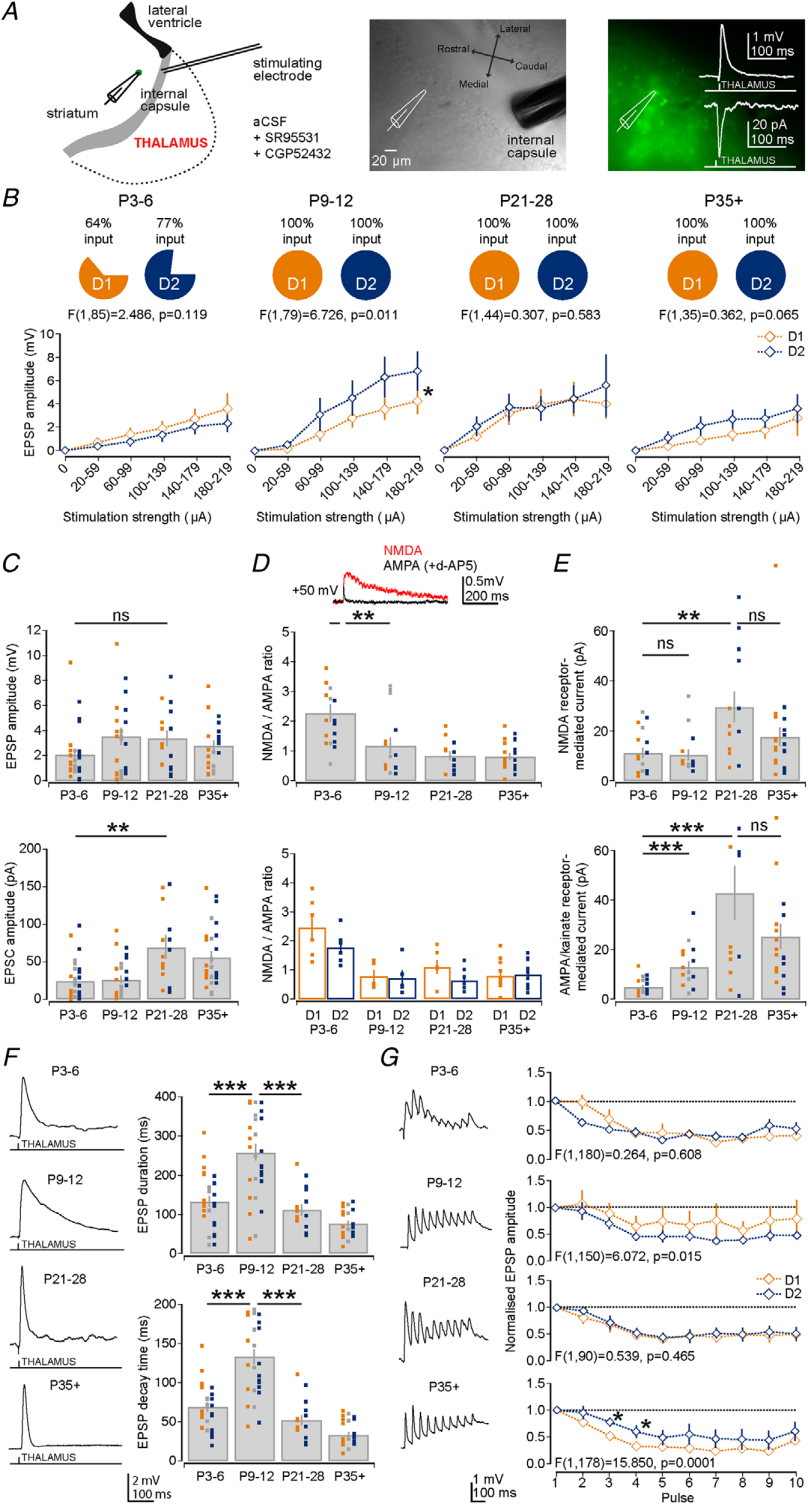
Rapid development of excitatory thalamic inputs onto D1 and D2 SPNs *A*, diagram of the recording configuration and placement of stimulation electrode in the internal capsule to activate thalamic afferents (left). Example Dodt contrast image (middle) and fluorescence image (right) of the striatum in a D2–GFP transgenic mouse. Inset: example of an evoked thalamic EPSP and EPSC. *B*, graphs of EPSP amplitude across a range of stimulation strengths (range 20–220 µA) for the different age ranges. Note the comparable amplitudes of evoked EPSPs in both the D1 (orange) and D2 (blue) SPNs, except at P9–12 when the D2 SPNs transiently receive a stronger thalamic input. Note that only ∼70% of SPNs exhibited a response at P3–6 whereas they always exhibited a response at later age ranges. *C*, bar plot of the maximum evoked EPSP amplitudes, which progressively become larger across development (top). Bar plots of EPSC amplitudes also shows an increase in the thalamic evoked excitatory current (bottom). *D*, bar plots of the NMDA/AMPA ratio across the age ranges. Note the significant decrease in the ratio from P3–6 to P9–12 (top), for both D1 and D2 SPNs (bottom). *E*, bar plots of the NMDA receptor‐mediated currents (top) and AMPA/kainate receptor‐mediated currents (bottom). Note the significant increase in the AMPA/kainate receptor‐mediated current from P3–6 to P9–12. *F*, bar plots of the EPSP duration (top) and EPSP decay time. Note the transient and significantly longer EPSP duration and decay time at P9–12. *G*, graphs of the EPSP amplitude across 10 stimulations at 20 Hz showing that thalamic synapses at D1 and D2 SPNs predominantly exhibit short‐term depression at all age ranges. D1 SPNs: orange squares; D2 SPNs: blue squares; and unclassified SPNs: grey squares.

**Figure 6:**
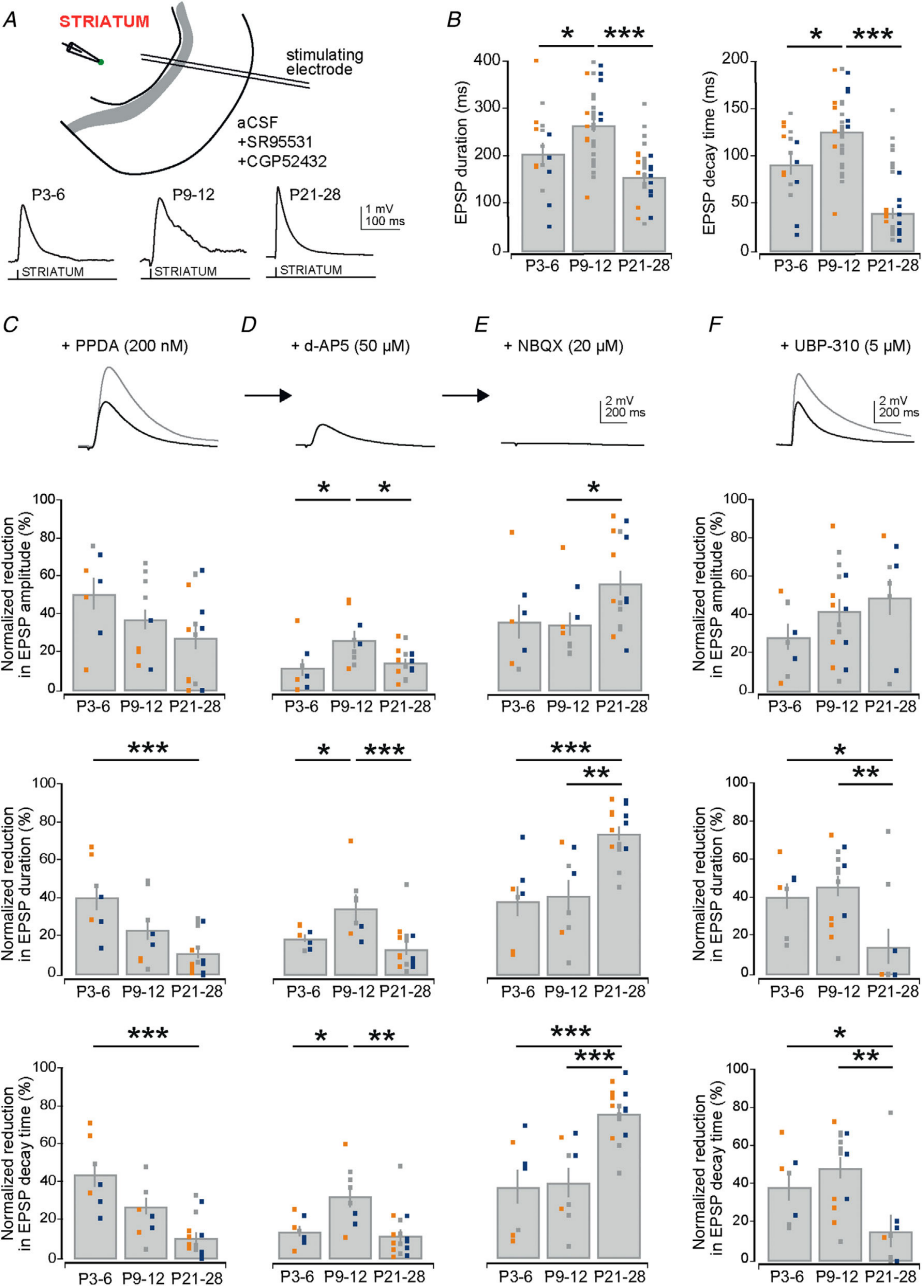
Changes in receptor expression at glutamatergic synapses onto striatal D1 and D2 SPNs *A*, diagram of recording configuration consisting of a stimulation electrode placed in the striatum close to the recorded SPNs (top). All recordings were performed in the presence of the GABA receptor antagonists. Example traces of striatal evoked EPSPs at three different age ranges (bottom). *B*, bar plots of the duration (left) and decay time (right) of striatal evoked EPSPs. Note the transient and significantly longer EPSP duration and decay time at P9–12. *C*, example trace and bar plots of the reduction of the normalized EPSP amplitude (top), duration (middle) and decay time (bottom) after superfusion of the NMDA receptor NR2C/D subunit‐selective antagonist PPDA (200 nm). Note the dominant effect on all parameters is at P3–6. *D*, example trace and bar plots of the reduction of the normalized EPSP amplitude (top), duration (middle) and decay time (bottom) after further addition of the NMDA receptor antagonist d‐AP5 (50 µm) to the superfusate. Note the dominant effect on all parameters is at P9–12. *E*, example trace and bar plots of the reduction of the normalized EPSP amplitude (top), duration (middle) and decay time (bottom) after final addition of the AMPA/kainate receptor antagonist NBQX (20 µm) to the superfusate, which fully blocks the residual EPSP. *F*, example trace and bar plots of the reduction of the normalized EPSP amplitude (top), duration (middle) and decay time (bottom) after addition of the kainate receptor antagonist UBP‐310 (5 µm) to the superfusate. Note that across development UBP‐310 exhibits an increasing effect on the amplitude of the EPSP and a decreasing effect on duration and decay time.

**Figure 7:**
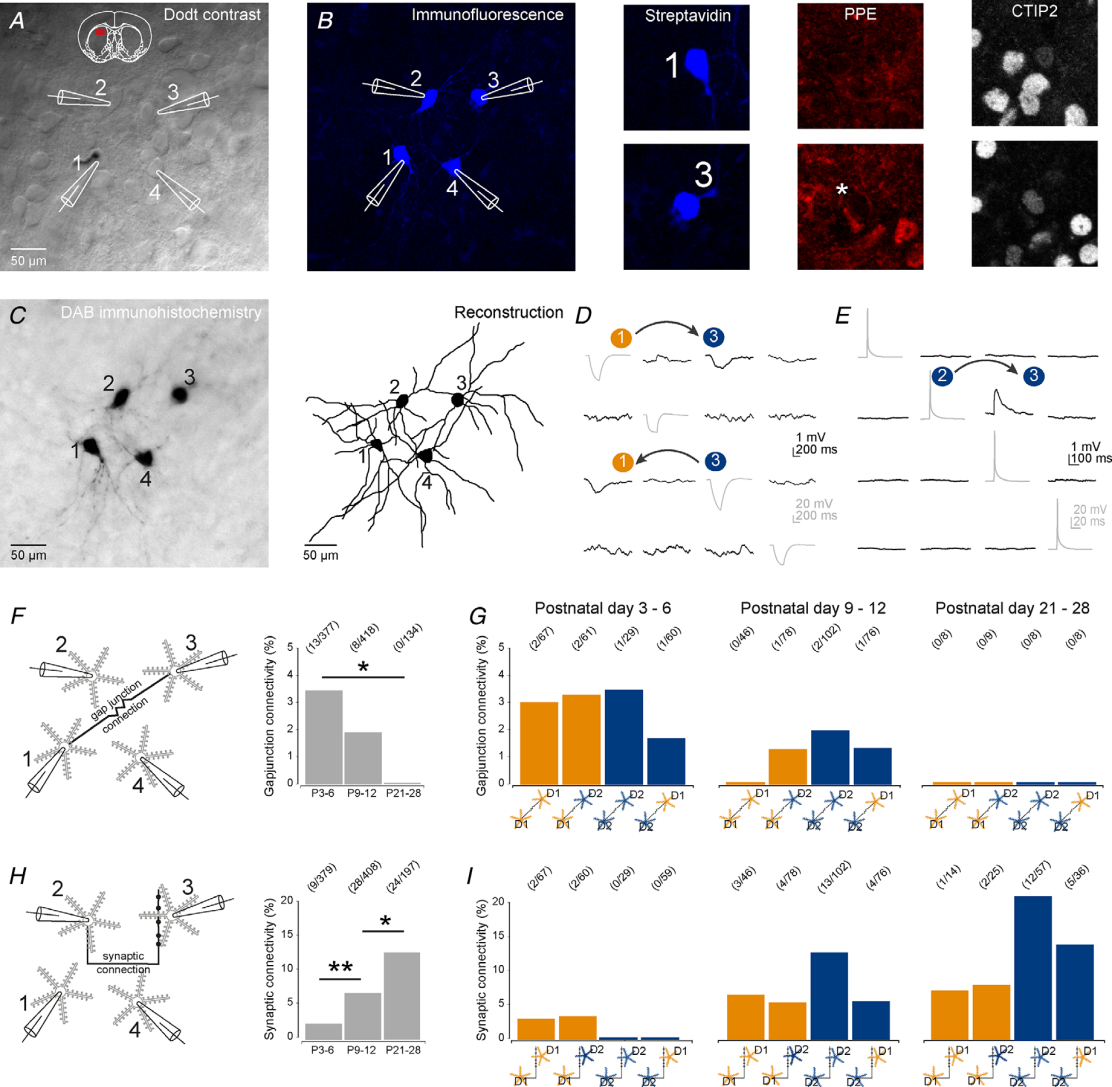
Gradual replacement of symmetrical gap junctions with precise inhibitory synaptic connections between SPNs *A*, Dodt‐contrast image of recording configuration consisting of four simultaneously patched SPNs. *B*, *post hoc* immunocytochemistry of recorded neurons using antibodies against streptavidin, PPE and CTIP2 allowed for classification of neurons as D1 or D2 SPNs. Note that SPN no. 1 is PPE negative and CTIP2 positive and therefore a D1 SPN, whereas SPN no. 2 is positive for PPE (indicated by asterisk) and therefore a D2 SPN. *C*, subsequently the slices were processed for DAB immunohistochemistry to label SPNs (left) and reveal dendritic structures allowing for reconstruction of SPNs (right). *D*, hyperpolarizing current steps revealed the presence of potential gap junctions connecting recorded SPNs. Note the presence of bidirectional gap junctions between D1 SPN no. 1 (orange) and D2 SPN no. 3 (blue). *E*, suprathreshold current injections elicited action potentials in recorded SPNs and revealed potential synaptic connections to other simultaneously recorded SPNs. Note the presence of a unidirectional synaptic connection from D2 SPN no. 2 to D2 SPN no. 3. *F*, diagram of experimental set‐up to test for potential gap junctions between SPNs (left). Bar plots showing a significant decrease in the incidence of detected gap junctions as the SPNs mature (right). *G*, bar plots of the incidence of gap junctions between D1 and D2 SPNs across the age ranges. Note the relatively uniform incidence of gap junctions in all SPN groups at P3–6 followed by a progressive reduction and absence of detected gap junctions at P21–28. *H*, diagram of experimental set‐up to test for synaptic connections between SPNs (left). Bar plots showing a progressive and significant increase in the incidence of detected synaptic connections as the SPNs mature (right). *I*, bar plots of incidences of synaptic connections between D1 and D2 SPNs across the age ranges. Note the earliest appearance of synaptic connections at P3–6 from D1 SPNs only. By P9–12 synaptic connections from both D1 and D2 SPNs can be observed and relative biases in synaptic connectivity, i.e. high incidence of connectivity between D2 SPN, are already apparent and are maintained.

All data are presented as means ± SEM; *n* refers to the number of neurons tested. The following numbers of animals were used for the datasets as reported in Fig. 1: P3–6: 28 animals; P9–12: 27 animals; P21–28: 46 animals; and P35+: 31 animals; Fig. 2: P3–6: 11 animals; P9–12: 9 animals; P21–28: 16 animals; and P35+: 13 animals; Fig. 3: P3–6: 5 animals; P9–12: 4 animals; P21–28: 6 animals; and P35+: 4 animals; Fig. 4: P3–6: 20 animals; P9–12: 25 animals; P21–28: 19 animals; and P35+: 18 animals; Fig. 5: P3–6: 27 animals; P9–12: 30 animals; P21–28: 17 animals; and P35+: 23 animals; Fig. 6: P3–6: 5 animals; P9–12: 5 animals; P21–28: 6 animals; Fig. 7: P3–6: 28 animals; P9–12: 27 animals; P21–28: 46 animals. Statistical tests were all two‐tailed and performed using SPSS Statistics 17.0 (SPSS Inc., Chicago, IL, USA, RRID:SCR_002865) or Prism version 5.0 (GraphPad Software Inc., La Jolla, CA, USA, RRID:SCR_002798). Gap junction and synaptic connection incidence were compared using Fisher's exact test. Continuous data were assessed for normality and appropriate parametric (ANOVA, Student's paired *t* test and unpaired *t* test) or non‐parametric (Mann–Whitney *U*) statistical tests were applied (^*^ *P* < 0.05, ^**^ *P* < 0.01, ^***^ *P* < 0.001).

## Results

### Gradual development of the electrophysiological properties of D1 and D2 SPNs

We first investigated the development of intrinsic electrophysiological properties of striatal D1 and D2 SPNs and their ability to generate action potentials using whole‐cell current‐clamp recordings of SPNs in mouse brain slices at postnatal day (P)3–6, P9–12, P21–28 and P35 and older (Fig. 1 *A*). Mice consisted of heterozygous D1 and D2–GFP mice on a C57/BL6 background as well as wild‐type C57/BL6 mice (see Methods) and we therefore always included biocytin in the intracellular solution, followed by immunocytochemistry for CTIP2 and PPE (see Methods) (Gerfen *et al*. 1990; Arlotta *et al*. 2008) to confirm, whenever possible, whether recordings were made from D1 or D2 SPNs (Fig. 1 *B*). Hyperpolarizing and depolarizing current steps were injected into the recorded SPNs to characterize their electrophysiological properties (Fig. 1 *C* and Table 1).

**Table 1:** Intrinsic membrane properties of D1 and D2 SPNs

	D1	D2	*P*	All
P3–6				
Resting membrane potential (mV)	−63.2 ± 0.9	−60.7 ± 0.8	**0.042**	−62.59 ± 0.5
Input resistance (MΩ)	1337.0 ± 96.3	1503.4 ± 100.7	0.240	1359.9 ± 52.6
Membrane time constant (ms)	50.1 ± 3.1	43.8 ± 3.0	0.164	47.3 ± 1.7
Membrane capacitance (pF)	6.1 ± 4.8	10.4 ± 4.7	0.792	10.4 ± 3.6
Spike threshold (mV)	−41.4 ± 0.7	−41.8 ± 0.8	0.658	−42.0 ± 0.4
Spike rate (50 pA) (Hz)	9.4 ± 1.1	9.9 ± 1.4	0.760	10.5 ± 0.7
Spike rate (40 pA) (Hz)	8.2 ± 1.0	8.6 ± 1.2	0.799	9.1 ± 0.6
Spike rate (30 pA) (Hz)	6.6 ± 0.8	7.3 ± 0.9	0.545	7.9 ± 0.6
Spike rate (20 pA) (Hz)	5.9 ± 0.8	6.8 ± 0.8	0.419	6.9 ± 0.5
Spike rate (10 pA) (Hz)	4.6 ± 1.0	3.9 ± 0.4	0.452	4.6 ± 0.4
First ISI (ms)	61.5 ± 5.8	61.5 ± 7.4	0.997	59.0 ± 3.2
Second ISI (ms)	63.3 ± 5.4	52.8 ± 4.4	0.163	58.5 ± 2.7
Third ISI (ms)	60.7 ± 6.3	58.1 ± 4.6	0.752	62.8 ± 3.3
Fourth ISI (ms)	61.4 ± 4.0	61.2 ± 4.4	0.985	61.4 ± 2.8
First spike amplitude (mV)	48.4 ± 2.5	40.7 ± 2.3	**0.028**	45.6 ± 1.4
Second spike amplitude (mV)	35.6 ± 3.1	25.7 ± 2.9	**0.023**	32.4 ± 1.7
First spike duration (ms)	4.6 ± 0.3	5.5 ± 0.3	**0.038**	4.8 ± 0.2
Second spike duration (ms)	6.4 ± 0.5	9.1 ± 1.0	**0.015**	7.1 ± 0.4
P9–12				
Resting membrane potential (mV)	−71.0 ± 0.9	−67.6 ± 0.8	**0.004**	−69.4 ± 0.5
Input resistance (MΩ)	407.8 ± 21.5	522.4 ± 25.4	**0.001**	485.0 ± 17.5
Membrane time constant (ms)	20.7 ± 1.0	24.2 ± 1.1	**0.029**	22.5 ± 0.7
Membrane capacitance (pF)	33.9 ± 7.1	25.4 ± 3.7	0.546	29.6 ± 4.0
Spike threshold (mV)	−44.3 ± 0.6	−45.4 ± 0.6	0.258	−44.9 ± 0.4
Spike rate (100 pA) (Hz)	15.7 ± 1.0	18.5 ± 0.8	**0.033**	16.5 ± 0.6
Spike rate (80 pA) (Hz)	13.6 ± 1.1	16.8 ± 0.9	**0.024**	15.1 ± 0.6
Spike rate (60 pA) (Hz)	13.0 ± 1.2	16.3 ± 0.9	**0.025**	14.4 ± 0.6
Spike rate (40 pA) (Hz)	11.0 ± 1.4	13.1 ± 1.0	0.263	11.4 ± 0.8
Spike rate (20 pA) (Hz)	6.8 ± 2.2	8.3 ± 1.8	0.615	8.5 ± 1.1
First ISI (ms)	59.3 ± 5.3	46.4 ± 3.5	**0.036**	50.8 ± 2.6
Second ISI (ms)	51.1 ± 3.6	41.2 ± 1.8	**0.007**	45.6 ± 1.6
Third ISI (ms)	54.3 ± 4.0	48.2 ± 2.1	0.141	51.2 ± 1.9
Fourth ISI (ms)	51.3 ± 2.4	55.2 ± 4.0	0.479	54.6 ± 2.5
First spike amplitude (mV)	69.5 ± 1.9	64.6 ± 1.8	0.066	67.3 ± 1.2
Second spike amplitude (mV)	60.1 ± 2.5	54.3 ± 1.8	0.052	56.0 ± 1.4
First spike duration (ms)	2.6 ± 0.1	2.5 ± 0.1	0.849	2.6 ± 0.1
Second spike duration (ms)	3.2 ± 0.1	3.6 ± 0.1	0.064	3.5 ± 0.1
P21–28				
Resting membrane potential (mV)	−82.2 ± 0.8	−78.4 ± 1.2	**0.013**	−79.1 ± 0.6
Input resistance (MΩ)	76.2 ± 4.7	100.7 ± 10.7	**0.049**	93.4 ± 4.6
Membrane time constant (ms)	2.5 ± 0.2	3.0 ± 0.2	0.138	2.8 ± 0.1
Membrane capacitance (pF)	40.4 ± 8.1	58.5 ± 8.2	0.177	48.6 ± 6.2
Spike threshold (mV)	−44.1 ± 1.2	−46.9 ± 1.3	0.116	−43.3 ± 0.7
Spike rate (500 pA) (Hz)	32.8 ± 2.2	36.4 ± 2.7	0.304	32.4 ± 1.4
Spike rate (400 pA) (Hz)	27.6 ± 2.4	34.2 ± 1.9	**0.041**	28.7 ± 1.2
Spike rate (300 pA) (Hz)	21.8 ± 2.1	30.3 ± 2.3	**0.009**	24.9 ± 1.2
Spike rate (200 pA) (Hz)	16.7 ± 1.9	22.3 ± 2.4	0.099	19.5 ± 1.2
Spike rate (100 pA) (Hz)	9.5 ± 2.2	14.1 ± 2.6	0.228	12.6 ± 1.5
First ISI (ms)	36.9 ± 8.2	18.9 ± 2.0	**0.048**	26.6 ± 2.5
Second ISI (ms)	33.4 ± 3.2	24.8 ± 3.5	0.078	28.5 ± 1.6
Third ISI (ms)	34.8 ± 3.0	30.8 ± 6.5	0.580	32.6 ± 1.9
Fourth ISI (ms)	34.3 ± 2.3	30.1 ± 2.5	0.232	33.4 ± 1.3
First spike amplitude (mV)	88.5 ± 3.2	79.0 ± 3.8	0.067	81.2 ± 2.0
Second spike amplitude (mV)	71.2 ± 5.8	62.9 ± 5.0	0.285	64.8 ± 2.4
First spike duration (ms)	2.0 ± 0.1	2.0 ± 0.1	0.741	2.0 ± 0.1
Second spike duration (ms)	2.7 ± 0.1	2.7 ± 0.2	0.749	2.6 ± 0.2
P35+				
Resting membrane potential (mV)	−80.5 ± 1.0	−78.0 ± 1.1	0.110	−80.0 ± 0.6
Input resistance (MΩ)	86.4 ± 8.2	112.6 ± 14.0	0.100	91.3 ± 5.7
Membrane time constant (ms)	2.6 ± 0.6	3.1 ± 0.4	0.458	2.6 ± 0.2
Membrane capacitance (pF)	118.5 ± 36.4	139.1 ± 63.2	0.999	127.7 ± 33.1
Spike threshold (mV)	−39.8 ± 2.2	−42.3 ± 2.4	0.452	−42.3 ± 1.0
Spike rate (500 pA) (Hz)	32.3 ± 7.1	43.3 ± 2.8	0.103	33.8 ± 2.0
Spike rate (400 pA) (Hz)	30.0 ± 5.9	40.5 ± 1.8	**0.048**	31.1 ± 1.9
Spike rate (300 pA) (Hz)	24.0 ± 5.9	33.3 ± 1.9	0.090	25.9 ± 1.9
Spike rate (200 pA) (Hz)	20.3 ± 6.7	20.6 ± 3.0	0.963	19.5 ± 1.9
Spike rate (100 pA) (Hz)	13.8 ± 3.8	10.0 ± 7.5	0.698	10.3 ± 1.9
First ISI (ms)	20.1 ± 3.8	20.4 ± 2.6	0.947	24.4 ± 2.1
Second ISI (ms)	21.5 ± 3.4	20.6 ± 1.3	0.782	25.3 ± 1.8
Third ISI (ms)	22.5 ± 3.1	20.4 ± 1.2	0.486	29.3 ± 2.1
Fourth ISI (ms)	24.7 ± 3.3	21.4 ± 1.3	0.298	31.9 ± 2.3
First spike amplitude (mV)	71.0 ± 6.9	71.8 ± 7.9	0.869	82.6 ± 3.4
Second spike amplitude (mV)	59.2 ± 6.6	66.1 ± 5.2	0.417	70.1 ± 3.0
First spike duration (ms)	2.5 ± 1.0	1.9 ± 0.2	0.465	1.8 ± 0.1
Second spike duration (ms)	1.9 ± 0.6	2.3 ± 0.3	0.531	2.3 ± 0.2

Data are given as means ± SEM, statistical comparisons by Mann–Whitney *U* and Student's *t* test. *P*‐values shown in bold are statistically significant.

Firstly, we found that in the earliest postnatal period (P3–6) most SPNs (83.0%) were already able to initiate small amplitude action potentials (Fig. 1 *C*). The action potentials of D1 SPNs exhibited subtle but significant maturational differences, consistent with their suggested earlier birthdate (Marchand & Lajoie, 1986; van der Kooy & Fishell, 1987; Kelly *et al*. 2018), including relatively larger and narrower action potentials (Table 1). These differences were transient and in later stages all SPNs generated large amplitude and narrow action potentials (Table 1). We found we could elicit higher frequencies of action potentials as they matured (D1: P3–6: 8.2 ± 1.0 Hz; P9–12 13.6 ± 1.1 Hz; P21–28: 27.6 ± 2.4 Hz; and P35+: 30.0 ± 5.9 Hz; P3–6 *vs*. P9–12: *P* = 0.00043; P9–12 *vs*. P21–28: *P* < 0.0001; and P21–28 *vs*. P35+: *P* = 0.59; Mann–Whitney *U* test; and D2; P3–6: 8.6 ± 1.2 Hz; P9–12 16.8 ± 0.9 Hz; P21–28: 34.2 ± 1.9 Hz; and P35+: 40.5 ± 1.8 Hz; P3–6 *vs*. P9–12: *P* < 0.0001; P9–12 *vs*. P21–28: *P* < 0.0001; and P21–28 *vs*. P35+: *P* = 0.014; Mann–Whitney *U* test; Fig. 1 *C* and Table 1). Interestingly, the D2 SPNs exhibited consistently higher firing frequencies (P9–12 at 80 pA: D1: 13.6 ± 1.1 Hz and D2: 16.8 ± 0.9 Hz; P21–28 at 400 pA: D1: 27.6 ± 2.4 Hz and D2: 34.2 ± 1.9 Hz; P35+ at 400 pA: D1: 30.0 ± 5.9 Hz and D2: 40.5 ± 1.8 Hz; *P* = 0.024, *P* = 0.041 and *P* = 0.057, *t* test; Fig. 1 *C* and Table 1) in the second and later postnatal weeks.

Secondly, both D1 and D2 SPNs exhibited a progressive maturation of their intrinsic membrane properties, including the emergence of a pronounced inward rectifying current at later developmental stages (Fig. 1 *D*), a more hyperpolarized membrane potential (Fig. 1 *E*) and a lowering of input resistance (Fig. 1 *F*). The higher action potential frequency seen in D2 SPNs might well result from their consistently more depolarized membrane potential (P9–12: D1: −71.0 ± 0.9 mV and D2: −67.6 ± 0.8 mV, *P* = 0.004; P21–28: D1: −82.2 ± 0.8 mV and D2: −78.4 ± 1.2 mV, *P* = 0.013; and P35+: D1: −80.5 ± 1.0 mV and D2: −78.0 ± 1.1 mV, *P* = 0.110; *t* test; Fig. 1 *E*) and higher input resistance (P9–12: D1: 407.8 ± 21.5 MΩ and D2: 522.4 ± 25.4 MΩ, *P* = 0.001; P21–28: D1: 76.2 ± 4.7 MΩ and D2: 100.7 ± 10.7 MΩ, *P* = 0.049; and P35+: D1: 86.4 ± 8.2 MΩ and D2: 112.6 ± 14.0 MΩ, *P* = 0.100; *t* test; Fig. 1 *F*).

In conclusion, we found that most D1 and D2 SPNs are able to generate action potentials shortly after birth and many of their electrophysiological properties develop in parallel. However, maturational differences could be seen early on, including narrower and larger action potentials in the D1 SPNs. Furthermore, significant differences in the intrinsic membrane properties were already observed in the first and second postnatal weeks, which persisted into adulthood, including a greater excitability and action potential frequency of D2 SPNs.

### Parallel development of dendrites and spines of D1 and D2 SPNs

We next investigated the development of the dendritic arbour of SPNs enabling them to receive and process excitatory and inhibitory synaptic inputs. The addition of biocytin in internal recording solutions allowed for DAB immunohistochemistry and reconstruction of previously recorded neurons (Fig. 2 *A*). We found a pronounced increase in the dendritic length of both D1 and D2 SPNs, which more than doubled (from ∼700 µm to ∼1800 µm) in the first 3 postnatal weeks (D1: P3–6: 698.4 ± 97.6 µm; P9–12: 1556.1 ± 181.6 µm; and P21–28: 2040.4 ± 328.4 µm; *P* = 0.00022 and *P* = 0.193, Mann–Whitney *U* test, *n* = 21, 10 and 7; and D2: P3–6: 757.4 ± 119.0 µm; P9–12: 1350.1 ± 83.9 µm; and P21–28: 2003.9 ± 248.9 µm; *P* = 0.00088 and *P* = 0.03, Mann–Whitney *U* test, *n* = 22, 13 and 9; Fig. 2 *B*) after which it did not seem to significantly increase further (D1, P35+: 2180.0 ± 211.6 µm and D2, P35+: 2162.3 ± 135.7 µm, *n* = 9 and *n* = 19; D1, P21–28 *vs*. P35+: *P* = 0.758 and D2 P21–28 *vs*. P35+: *P* = 0.809). This early developmental increase in dendritic arbour was concomitant with a significant increase in distal dendritic complexity allowing the SPNs to sample larger and extensive regions of striatum as revealed using Scholl analysis (P3–6 *vs*. P9–12: *F*(17, 691) = 8.98, *P* = 2.85 × 10^−21^; P9–12 *vs*. P21–28: *F*(20, 533) = 2.369, *P* = 0.00077; and P21–28 *vs*. P35+: *F*(20, 740) = 0.464, *P* = 0.979; ANOVA; Fig. 2 *C*). Both the increase in their dendritic arbour (Fig. 2 *B*) and in their dendritic complexity (Fig. 2 *C*) occurred in parallel for the D1 and D2 SPNs (dendritic arbour: P3–6: *P* = 0.827; P9–12: *P* = 0.738; P21–28: *P* = 0.758; and P35+: *P* = 0.980; Mann–Whitney *U* test; and dendritic complexity: P3–6: *P* = 0.816; P9–12: *P* = 0.091; P21–28: *P* = 0.827; and P35+: *P* = 0.971; ANOVA). One of the distinguishing features of mature SPNs is the radial orientation of their dendrites. We found that the orientation of the dendritic branches of both D1 and D2 SPNs exhibited this radial morphology from birth onwards, without obvious changes in the orientation of the dendritic arbour (Callaway & Borrell, 2011), and exhibiting a slight bias towards a lateral–ventral to medial–dorsal orientation (Fig. 2 *A* and *D*)

Synaptic inputs onto SPNs can be made on both the dendrites directly or on the dendritic spines (Somogyi *et al*. 1981; Bolam & Izzo, 1988; Yung *et al*. 1996; Doig *et al*. 2010). To characterize the development of spines, we investigated the density of dendritic spines on primary, secondary and tertiary dendritic branches on both D1 and D2 SPNs in the four age ranges (Fig. 2 *E*). Whereas we saw a significant increase in the average spine density (per µm of dendrite) as the neurons matured during the early postnatal periods (D1: P3–6: 0.13 ± 0.01; P9–12: 0.19 ± 0.02; P21–28: 0.35 ± 0.01; and P35+: 0.27 ± 0.02; *P* = 0.02, *P* = 0.004 and *P* = 0.01, Mann–Whitney *U* test, *n* = 8, 8, 4 and 6; and D2: P3–6: 0.13 ± 0.02; P9–12: 0.18 ± 0.01; P21–28: 0.26 ± 0.02; and P35+: 0.28 ± 0.02; *P* = 0.008, *P* = 0.04 and *P* = 0.5, Mann–Whitney *U* test, *n* = 10, 11, 3 and 15), this occurred in parallel for both D1 and D2 SPNs (*P* > 0.05).

In conclusion, we found that the general morphology of the D1 and D2 SPNs develops in parallel with similar increases in their dendritic arbour and spine density.

### Maturation of excitatory and inhibitory synaptic inputs onto D1 and D2 SPNs

Our results so far suggest that both D1 and D2 SPNs can already generate action potentials during the first postnatal week, and that their dendritic arbour and spine density develop mostly in parallel, allowing them to sample excitatory and inhibitory synaptic inputs from nearby axons. We next asked when synaptic inputs on SPNs are functional by performing whole‐cell voltage‐clamp recordings of SPNs in the presence of TTX (1 µm) at the four age ranges. This allowed for recordings of both spontaneous mEPSCs by holding the SPN membrane voltage at −70 mV (Fig. 3 *A*) and spontaneous mIPSCs by holding the SPN membrane voltage at 0 mV (Fig. 3 *D*). We confirmed that spontaneous miniature events could be blocked using respectively the AMPA/kainate receptor antagonist NBQX (10 µm) and the GABA_A_ receptor antagonist SR95531 (200 nm) (Fig. 3 *A* and *D*). Our first observation was that excitatory mEPSCs could be detected as early as P3–6 in both D1 and D2 SPNs (Fig. 3 *A* and *B*), which increased slightly in frequency but was already close to that seen in adulthood (∼1 Hz) (P3–6: 0.79 ± 0.10 Hz; P9–12: 0.88 ± 0.07 Hz; P21–28: 1.05 ± 0.10 Hz; and P35+: 0.89 ± 0.10 Hz; P3–6 *vs*. P35+ *P* = 0.518, Mann–Whitney *U* test, *n* = 15, 22, 17 and 14; Fig. 3 *B*). In contrast, the mEPSC amplitude exhibited a significant increase for both D1 and D2 SPNs from P3–6 to P9–12 (D1: P3–6: 3.06 ± 0.50 pA to P9–12: 7.13 ± 0.57 pA; and D2: P3–6: 2.52 ± 0.40 pA to P9–12: 6.67 ± 0.47 pA; *P* = 0.001 and *P* = 0.001, Mann–Whitney *U* test, both *n* = 6 and *n* = 7; Fig. 3 *C*) after which it remained constant. Importantly, no significant differences were found in either the mEPSC frequency or the mEPSC amplitude between the D1 and D2 SPNs at any of the age ranges investigated (all *P* > 0.05). These results suggest that excitatory synaptic inputs on SPNs are present and functional soon after birth and develop in parallel and similarly innervate both D1 and D2 SPNs with postsynaptic changes occurring between P3–6 and P9–12.

The responses of SPNs to excitatory inputs are modulated by concurrent inhibitory inputs that they might receive. We next investigated the development of inhibitory synaptic inputs onto SPNs as reflected in the frequency and amplitude of mIPSCs. We found that both D1 and D2 SPNs received inhibitory synaptic inputs starting from P3–6 onwards (Fig. 3 *D* and *E*). However, the frequency of the mIPSCs at this age range was comparatively low at ∼0.1 Hz, and exhibited a progressive and steady increase throughout the early age ranges (D1: P3–6: 0.12 ± 0.02 Hz; P9–12: 0.30 ± 0.05 Hz; P21–28: 0.63 ± 0.06 Hz; and P35+: 0.43 ± 0.07 Hz; *P* = 0.005, *P* = 0.008 and *P* = 0.051, Mann–Whitney *U* test, *n* = 6, 7, 6 and 7; and D2: P3–6: 0.12 ± 0.02 Hz; P9–12: 0.26 ± 0.03 Hz; P21–28: 0.71 ± 0.16 Hz; and P35+: 0.47 ± 0.09 Hz; *P* = 0.018, *P* = 0.012 and *P* = 0.19, Mann–Whitney *U* test, *n* = 6, 7, 4 and 5; Fig. 3 *E*) after which it dropped slightly (*P* > 0.05). Similar to the observations of the mEPSC amplitude, we found that the mIPSC amplitude also exhibited a significant increase from P3–6 to P9–12 (D1: P3–6: 3.03 ± 0.46 pA and P9–12: 8.09 ± 0.58 pA; and D2: P3–6: 2.94 ± 0.66 pA and P9–12: 7.61 ± 0.48 pA; *P* = 0.001 and *P* = 0.002, Mann–Whitney *U* test, both *n* = 5 and *n* = 7; Fig. 3 *F*) after which it did not significantly increase further.

These results suggest that functional excitatory and inhibitory synaptic inputs are present during the first postnatal week and are sampled by both D1 and D2 SPNs. Moreover, they suggest that between the first and second postnatal weeks, substantial postsynaptic changes occur as reflected in the greater mEPSC and mIPSC amplitudes. Lastly, whereas the mEPSC frequency stayed relatively constant, a progressive and steady increase in mIPSC frequency was seen implying a prolonged maturation of inhibitory inputs.

### Maturation of long‐range cortical excitatory inputs on striatal D1 and D2 SPNs

The main excitatory afferents to striatal SPNs arise from neurons located in the cortex and thalamus (Kemp & Powell, 1971; Buchwald *et al*. 1973; Smith *et al*. 2004). Although, cortical and thalamic axons and synapses have been shown to be present in the striatum early on in development (Nakamura *et al*. 2005; Sohur *et al*. 2014), it is currently unknown when and to what extent these inputs are sampled by the D1 and D2 SPNs. To investigate inputs coming from cortex we performed whole‐cell patch‐clamp recordings of SPNs in the dorsal striatum and activated excitatory afferents coming from cortex by giving single and trains of stimulation via a tungsten bipolar electrode placed in the external capsule (Fig. 4 *A*). These experiments were performed in the presence of GABA receptor antagonists to avoid erroneous activation of inhibitory inputs. Firstly, we found that not all D1 and D2 SPNs at P3–6 received cortical excitatory synaptic inputs (P3–6: D1: 75% and D2: 71%, *n* = 12 and 14) whereas those at P9–12 and older all did (Fig. 4 *B*). Across a wide range of stimulation strengths (range 20–220 µA), both the D1 and D2 SPNs received similar amplitude EPSPs (P3–6: *F*(1, 83) = 0.012, *P* = 0.918; P9–12: *F*(1, 94) = 0.262, *P* = 0.610; P21–28: *F*(1, 36) = 0.049, *P* = 0.826; and P35+: *F*(1, 50) = 0.409, *P* = 0.525; Fig. 4 *B*) with a mean synaptic delay of ∼4 ms (P3–6: 4.13 ± 0.34 ms; P9–12: 4.02 ± 0.33 ms; P21–28: 3.01 ± 0.50 ms; and P35+: 3.53 ± 0.90 ms). Indeed, comparing the maximum responses that we could elicit in SPNs across the age ranges showed only a modest developmental increase in EPSP amplitude (P3–6: 2.86 ± 0.64 mV; P9–12: 3.42 ± 0.66 mV; P21–28: 3.46 ± 0.62 mV; and P35+: 3.06 ± 0.64 mV; P3–6 *vs*. P21–28, *P* = 0.425, Mann–Whitney *U* test, *n* = 19, 26, 18 and 11; Fig. 4 *C*), but a more pronounced increase in EPSC amplitude (P3–6: 24.10 ± 6.61 pA; P9–12: 27.27 ± 5.25 pA; P21–28: 49.76 ± 9.23 pA; and P35+: 50.92 ± 12.13 pA; P3–6 *vs*. P21–28, *P* = 0.027, Mann–Whitney *U* test, *n* = 14, 19, 20 and 19; Fig. 4 *C*), which suggests a strengthening of corticostriatal synapses across development.

To investigate whether the observed increase in amplitude of the evoked excitatory response could be the result of changes in postsynaptic glutamate receptors, we analysed the contribution of both NMDA and AMPA/kainate glutamate receptors to the cortically evoked excitatory responses at the different age ranges. Evoked excitatory events were recorded from SPNs in whole‐cell voltage‐clamp mode at a holding potential of +50 mV and consisted of combined NMDA and AMPA/kainate receptor‐mediated currents (Fig. 4 *D*). After baseline recording, the NMDA receptor antagonist d‐AP5 (50 µm) was superfused thereby isolating the AMPA/kainate receptor‐mediated current. Analysis of the ratio of peak amplitude NMDA and AMPA/kainate receptor‐mediated currents revealed a decline in the NMDA/AMPA ratio across early development, especially evident from P9–12 to P21–28 (P3–6: 2.48 ± 0.49; P9–12: 2.15 ± 0.30; P21–28: 1.00 ± 0.39; and P35+: 1.43 ± 0.21; P3–6 *vs*. P21–28: *P* = 0.004, Mann–Whitney *U* test, *n* = 16, 20, 12 and 11; Fig. 4 *D*) and similarly for both the D1 and D2 SPNs (Fig. 4 *D* and Table 2).

**Table 2:** Properties of excitatory cortical synapses onto D1 and D2 SPNs

	D1	D2	*P*	All
P3–6				
EPSP amplitude (mV)	3.13 ± 1.06	2.62 ± 0.80	0.905	2.86 ± 0.64
EPSP duration (ms)	167.88 ± 40.84	160.61 ± 18.90	0.654	164.07 ± 21.25
EPSP rise time (ms)	6.71 ± 0.80	8.10 ± 1.20	0.387	7.44 ± 0.74
EPSP decay time (ms)	90.28 ± 17.80	72.83 ± 9.09	0.705	81.14 ± 9.66
Short term plasticity (2 *vs*. 1)	0.81 ± 0.23	0.94 ± 0.21	0.682	0.86 ± 0.15
Short term plasticity (3 *vs*. 1)	0.61 ± 0.11	0.69 ± 0.11	0.642	0.64 ± 0.07
Short term plasticity (4 *vs*. 1)	0.58 ± 0.19	0.48 ± 0.10	0.686	0.52 ± 0.11
Short term plasticity (5 *vs*. 1)	0.78 ± 0.26	0.31 ± 0.04	0.099	0.55 ± 0.14
Short term plasticity (6 *vs*. 1)	0.67 ± 0.22	0.47 ± 0.13	0.458	0.59 ± 0.13
EPSC amplitude (pA)	22.79 ± 7.55	24.33 ± 12.58	0.432	24.01 ± 6.61
EPSC duration (ms)	28.22 ± 3.98	19.70 ± 2.47	0.106	23.25 ± 2.44
EPSC rise time (ms)	2.39 ± 0.32	1.76 ± 0.32	0.149	2.02 ± 0.24
EPSC decay time (ms)	11.67 ± 1.99	8.36 ± 1.01	0.268	9.74 ± 1.08
NMDA receptor current (pA)	19.42 ± 4.73	11.28 ± 6.87	0.174	14.84 ± 2.57
AMPA receptor current (pA)	8.82 ± 2.31	8.97 ± 8.46	0.758	8.90 ± 1.82
NMDA/AMPA ratio	2.50 ± 0.37	2.46 ± 0.85	0.470	2.48 ± 0.49
P9–12				
EPSP amplitude (mV)	3.01 ± 0.80	3.16 ± 0.89	0.979	3.42 ± 0.66
EPSP duration (ms)	214.62 ± 18.13	188.91 ± 16.04	0.205	203.41 ± 11.12
EPSP rise time (ms)	6.55 ± 0.71	7.83 ± 0.93	0.230	7.16 ± 0.52
EPSP decay time (ms)	115.58 ± 10.97	90.55 ± 7.76	0.056	103.17 ± 6.42
Short term plasticity (2 *vs*. 1)	0.95 ± 0.13	0.72 ± 0.09	0.169	0.84 ± 0.08
Short term plasticity (3 *vs*. 1)	0.74 ± 0.13	0.65 ± 0.15	0.665	0.68 ± 0.09
Short term plasticity (4 *vs*. 1)	0.46 ± 0.08	0.40 ± 0.07	0.574	0.42 ± 0.05
Short term plasticity (5 *vs*. 1)	0.48 ± 0.08	0.39 ± 0.10	0.451	0.43 ± 0.06
Short term plasticity (6 *vs*. 1)	0.42 ± 0.07	0.47 ± 0.12	0.712	0.44 ± 0.06
EPSC amplitude (pA)	31.62 ± 12.23	24.73 ± 4.71	0.711	27.27 ± 5.25
EPSC duration (ms)	43.87 ± 11.80	33.91 ± 3.34	0.964	40.00 ± 7.28
EPSC rise time (ms)	3.43 ± 1.33	2.78 ± 0.46	0.536	3.17 ± 0.82
EPSC decay time (ms)	18.43 ± 4.64	14.28 ± 1.57	0.930	16.82 ± 2.88
NMDA receptor current (pA)	22.09 ± 4.69	11.47 ± 4.78	**0.035**	15.93 ± 3.36
AMPA receptor current (pA)	10.33 ± 2.20	5.90 ± 1.43	0.133	7.79 ± 1.28
NMDA/AMPA ratio	2.33 ± 0.36	2.14 ± 0.50	0.720	2.15 ± 0.30
P21–28				
EPSP amplitude (mV)	3.01 ± 1.07	4.24 ± 0.79	0.210	3.46 ± 0.62
EPSP duration (ms)	99.92 ± 9.06	133.04 ± 25.01	0.515	112.81 ± 11.76
EPSP rise time (ms)	5.34 ± 0.56	6.00 ± 0.73	0.515	5.47 ± 0.39
EPSP decay time (ms)	45.72 ± 4.35	64.50 ± 12.30	0.360	55.86 ± 5.90
Short term plasticity (2 *vs*. 1)	1.06 ± 0.40	0.99 ± 0.16	0.886	0.98 ± 0.17
Short term plasticity (3 *vs*. 1)	1.24 ± 0.46	0.94 ± 0.19	0.550	1.05 ± 0.20
Short term plasticity (4 *vs*. 1)	0.84 ± 0.31	0.49 ± 0.10	0.301	0.63 ± 0.14
Short term plasticity (5 *vs*. 1)	0.67 ± 0.27	0.54 ± 0.17	0.703	0.57 ± 0.13
Short term plasticity (6 *vs*. 1)	1.35 ± 0.72	0.79 ± 0.26	0.476	0.95 ± 0.31
EPSC amplitude (pA)	46.69 ± 14.35	57.27 ± 16.43	0.673	49.76 ± 9.23
EPSC duration (ms)	30.30 ± 2.26	24.69 ± 2.43	0.252	26.51 ± 1.51
EPSC rise time (ms)	2.26 ± 0.38	1.59 ± 0.16	0.114	1.88 ± 0.16
EPSC decay time (ms)	12.94 ± 0.92	10.79 ± 1.19	0.174	11.36 ± 0.68
NMDA receptor current (pA)	16.65 ± 4.21	10.01 ± 2.77	0.343	13.88 ± 2.79
AMPA receptor current (pA)	30.41 ± 13.27	15.32 ± 2.91	0.935	24.12 ± 7.90
NMDA/AMPA ratio	1.23 ± 0.65	0.68 ± 0.21	0.876	1.00 ± 0.39
P35+				
EPSP amplitude (mV)	3.11 ± 1.13	3.28 ± 0.93	0.917	3.06 ± 0.64
EPSP duration (ms)	72.25 ± 13.48	97.07 ± 20.90	0.340	81.30 ± 10.89
EPSP rise time (ms)	4.33 ± 0.49	4.29 ± 0.53	0.711	4.37 ± 0.34
EPSP decay time (ms)	32.15 ± 6.32	44.65 ± 10.15	0.384	36.68 ± 5.20
Short term plasticity (2 *vs*. 1)	1.16 ± 0.20	0.84 ± 0.09	0.129	0.01 ± 0.07
Short term plasticity (3 *vs*. 1)	0.91 ± 0.21	0.73 ± 0.07	0.422	0.79 ± 0.07
Short term plasticity (4 *vs*. 1)	0.73 ± 0.16	0.62 ± 0.06	0.522	0.71 ± 0.07
Short term plasticity (5 *vs*. 1)	0.73 ± 0.16	0.56 ± 0.07	0.329	0.61 ± 0.06
Short term plasticity (6 *vs*. 1)	0.66 ± 0.10	0.62 ± 0.09	0.783	0.65 ± 0.05
EPSC amplitude (pA)	20.00 ± 16.43	25.45 ± 9.69	0.931	50.92 ± 12.13
EPSC duration (ms)	24.24 ± 6.64	31.50 ± 3.98	0.421	30.14 ± 4.01
EPSC rise time (ms)	2.71 ± 0.50	2.87 ± 0.39	0.548	2.67 ± 0.24
EPSC decay time (ms)	9.70 ± 2.95	15.12 ± 2.18	0.095	12.17 ± 1.67
NMDA receptor current (pA)	32.33 ± 13.95	21.08 ± 5.54	0.886	23.60 ± 5.66
AMPA receptor current (pA)	27.03 ± 6.17	20.81 ± 6.40	0.486	20.14 ± 3.68
NMDA/AMPA ratio	1.30 ± 0.27	1.11 ± 0.22	0.730	1.43 ± 0.21

Data are given as means ± SEM, statistical comparisons Mann–Whitney *U* and *t* tests. *P*‐values shown in bold are statistically significant.

This change in the NMDA/AMPA ratio resulted from a pronounced increase in AMPA receptor‐mediated current from P9–12 to P21–28 (P3–6: 8.90 ± 1.82 pA; P9–12: 7.79 ± 1.28 pA; P21–28: 24.12 ± 7.90 pA; and P35+: 20.14 ± 3.68 pA; P9–12 *vs*. P21–28: *P* = 0.023, Mann–Whitney *U* test, *n* = 16, 20, 12 and 11), whereas the NMDA receptor‐mediated current did not change significantly (P3–6: 14.84 ± 2.57 pA; P9–12: 15.93 ± 3.36 pA; P21–28: 13.88 ± 2.79 pA; and P35+: 23.60 ± 5.66 pA; P9–12 *vs*. P21–28: 0.945, Mann–Whitney *U* test, *n* = 16, 20, 12 and 11; Fig. 4 *E*). Interestingly, the D1 SPNs received a significantly larger NMDA receptor‐mediated current at P9–12 compared to D2 SPNs (D1: 22.09 ± 4.69 pA and D2: 11.47 ± 4.78 pA; *P* = 0.035, Mann–Whitney *U* test, *n* = 9 and *n* = 9; Table 2), which was also apparent at P3–6 (D1: 19.42 ± 4.73 pA and D2: 11.28 ± 6.87 pA; Table 2).

Such changes in postsynaptic glutamate receptor types are predicted to change the EPSP kinetics (Seeburg, 1993). Indeed, we found that both the EPSP duration and the EPSP decay time were highly dynamic across the age ranges (Fig. 4 *F* and Table 2) with a transient phase at P9–12 when the EPSP duration (P3–6: 164.07 ± 21.25 ms; P9–12: 203.41 ± 11.12 ms; P21–28: 112.81 ± 11.76 ms; and P35+: 81.30 ± 10.89 ms; P3–6 *vs*. P9–12: *P* = 0.038 and P9–12 *vs*. P21–28: *P* < 0.0001, Mann–Whitney *U* test *n* = 21, 40, 21 and 20) and decay time (P3–6: 81.14 ± 9.66 ms; P9–12: 103.17 ± 6.42 ms; P21–28: 55.86 ± 5.90 ms; and P35+: 36.68 ± 5.20 ms; P3–6 *vs*. P9–12: *P* = 0.042 and P9–12 *vs*. P21–28: *P* < 0.0001, Mann–Whitney *U* test, *n* = 21, 40, 21 and 20) were longer than at any other point in development (Fig. 4 *F* and Table 2). These developmental changes were also reflected in the kinetics of the EPSCs (Table 2; EPSC duration: P3–6 *vs*. P9–12: *P* = 0.012; and P9–12 *vs*. P21–28: *P* = 0.042; and EPSC decay time: P3–6 *vs*. P9–12: *P* = 0.0089; and P9–12 *vs*. P21–28: *P* = 0.070). Neither the EPSP nor the EPSC kinetics differed between the D1 and D2 SPNs (Table 2; all *P* > 0.05).

Lastly, we investigated whether there were also presynaptic developmental changes that occurred at corticostriatal synapses, which could affect the short‐term plastic properties of the cortical synapses onto SPNs. Using trains of electrical stimulation (10 pulses at 20 Hz) we found that corticostriatal synapses were consistently depressing at all developmental ages (Fig. 4 *G*).

Combined, these results suggest that the excitatory cortical synapses onto D1 and D2 SPNs are functional in the first postnatal week and mostly develop in parallel and become stronger across the postnatal weeks, mainly from P9–12 onwards through an increase in AMPA receptor‐mediated transmission. The notable exception is a larger NMDA receptor‐mediated current in D1 SPNs in the second postnatal week. Lastly, we found that corticostriatal excitatory responses exhibit both a long duration and decay time in the second postnatal week.

### Maturation of long‐range cortical thalamic inputs on striatal D1 and D2 SPNs

The second major excitatory input to the striatal SPNs comes from the thalamus (Doig *et al*. 2010; Ellender *et al*. 2013; Smith *et al*. 2014), whose inputs are thought to arrive comparatively earlier in development (Nakamura *et al*. 2005). To investigate the development of the excitatory inputs coming from thalamus, we performed whole‐cell patch‐clamp recordings of D1 and D2 SPNs in the dorsal striatum and activated excitatory afferents from the thalamus by giving single and trains of stimulation via a tungsten bipolar electrode placed in the internal capsule (Fig. 5 *A*). These experiments were performed in modified horizontal sections, to retain as much of the thalamostriatal projections as possible (Ding *et al*. 2008; Smeal *et al*. 2008), and in the presence of GABA receptor antagonists to avoid erroneous activation of inhibitory inputs. Similar to our observations for cortical inputs, not all D1 and D2 SPNs at P3–6 received thalamic inputs (D1: 64% and D2: 77%, *n* = 14 and 13), whereas those at P9–12 and older all did (Fig. 5 *B*). For all SPNs receiving thalamic input (mean synaptic delay of ∼4 ms; P3–6: 4.31 ± 0.35 ms; P9–12: 3.78 ± 0.25 ms; P21–28: 3.69 ± 0.30 ms; and P35+: 3.89 ± 0.54 ms) we found that across a wide range of stimulation strengths (range 20–220 µA) both D1 and D2 SPNs mostly received comparable amplitude EPSPs, with the notable exception of D2 SPNs, which transiently receive a larger thalamic excitatory input at P9–12 (*F*(1, 79) = 6.726, *P* = 0.011; Fig. 5 *B*). This was also reflected in larger amplitude EPSCs as recorded from D2 SPN at P9–12 (D1: 20.32 ± 7.72 pA and D2: 49.17 ± 6.67 pA, *P* = 0.013, Mann–Whitney *U* test, *n* = 14 and 9; Table 3). In this case and others, the distance between the stimulation electrode and recording electrode was kept constant for both D1 and D2 SPNs (D1: 809 ± 53 µm and D2: 794 ± 83 µm, *n* = 13 and 12). Combining over all SPNs a modest increase across early development in thalamic evoked EPSPs was observed (P3–6: 2.15 ± 0.43 mV; P9–12: 3.59 ± 0.68 mV; P21–28: 3.43 ± 0.64 mV; and P35+: 2.83 ± 0.42 mV; P3–6 *vs*. P21–28, *P* = 0.112, Mann–Whitney *U* test, *n* = 28, 29, 17 and 21; Fig. 5 *C*) and a more pronounced increase in EPSC amplitude (P3–6: 24.60 ± 4.61 pA; P9–12: 26.96 ± 4.74 pA; P21–28: 69.91 ± 15.88 pA; and P35+: 56.24 ± 8.21 pA; P3–6 *vs*. P21–28, *P* = 3.7 × 10^−5^, Mann–Whitney *U* test, *n* = 30, 33, 18 and 26; Fig. 5 *C*) suggesting a developmental strengthening of thalamostriatal synapses.

**Table 3:** Properties of excitatory thalamic synapses onto D1 and D2 SPNs

	D1	D2	*P*	All
P3–6				
EPSP amplitude (mV)	2.40 ± 0.92	2.29 ± 0.64	0.882	2.15 ± 0.43
EPSP duration (ms)	178.05 ± 21.92	108.35 ± 17.07	**0.027**	133.79 ± 13.28
EPSP rise time (ms)	7.07 ± 0.49	4.97 ± 0.41	**0.006**	5.55 ± 0.40
EPSP decay time (ms)	87.81 ± 10.11	55.35 ± 7.48	**0.016**	69.54 ± 5.87
Short term plasticity (2 *vs*. 1)	0.97 ± 0.14	0.60 ± 0.06	**0.029**	0.76 ± 0.08
Short term plasticity (3 *vs*. 1)	0.66 ± 0.18	0.47 ± 0.06	0.330	0.62 ± 0.09
Short term plasticity (4 *vs*. 1)	0.40 ± 0.09	0.42 ± 0.09	0.861	0.44 ± 0.07
Short term plasticity (5 *vs*. 1)	0.40 ± 0.17	0.27 ± 0.06	0.428	0.38 ± 0.08
Short term plasticity (6 *vs*. 1)	0.39 ± 0.08	0.38 ± 0.07	0.936	0.42 ± 0.05
EPSC amplitude (pA)	17.76 ± 7.43	29.13 ± 9.24	0.332	24.60 ± 4.61
EPSC duration (ms)	25.82 ± 3.56	24.84 ± 3.21	0.796	28.13 ± 2.24
EPSC rise time (ms)	2.68 ± 0.45	1.73 ± 0.11	0.123	2.21 ± 0.20
EPSC decay time (ms)	14.04 ± 4.33	10.39 ± 1.34	0.796	13.37 ± 1.86
NMDA receptor current (pA)	10.70 ± 3.33	10.56 ± 3.59	0.937	11.25 ± 2.12
AMPA receptor current (pA)	4.38 ± 1.85	5.24 ± 1.18	0.589	5.05 ± 0.80
NMDA/AMPA ratio	2.47 ± 0.42	1.78 ± 0.23	0.241	2.28 ± 0.29
P9–12				
EPSP amplitude (mV)	3.37 ± 1.05	5.11 ± 1.52	0.356	3.59 ± 0.68
EPSP duration (ms)	255.23 ± 41.59	250.61 ± 28.09	0.853	259.13 ± 21.05
EPSP rise time (ms)	7.93 ± 1.56	6.23 ± 0.54	0.529	6.83 ± 0.64
EPSP decay time (ms)	132.81 ± 18.65	120.10 ± 14.09	0.604	133.98 ± 9.37
Short term plasticity (2 *vs*. 1)	1.07 ± 0.25	0.94 ± 0.17	0.675	1.03 ± 0.13
Short term plasticity (3 *vs*. 1)	0.89 ± 0.20	0.71 ± 0.11	0.478	0.76 ± 0.08
Short term plasticity (4 *vs*. 1)	0.65 ± 0.20	0.46 ± 0.08	0.397	0.59 ± 0.09
Short term plasticity (5 *vs*. 1)	0.75 ± 0.26	0.46 ± 0.11	0.340	0.58 ± 0.11
Short term plasticity (6 *vs*. 1)	0.67 ± 0.27	0.46 ± 0.09	0.491	0.53 ± 0.10
EPSC amplitude (pA)	20.32 ± 7.72	49.17 ± 6.67	**0.013**	26.96 ± 4.74
EPSC duration (ms)	45.86 ± 7.83	42.32 ± 4.35	0.657	44.77 ± 4.98
EPSC rise time (ms)	2.73 ± 0.37	2.11 ± 0.20	0.351	2.54 ± 0.23
EPSC decay time (ms)	20.22 ± 4.06	19.06 ± 2.09	0.492	20.05 ± 2.42
NMDA receptor current (pA)	8.08 ± 0.91	8.08 ± 2.25	0.421	10.42 ± 2.14
AMPA receptor current (pA)	12.84 ± 2.56	16.39 ± 5.36	0.841	13.18 ± 2.24
NMDA/AMPA ratio	0.78 ± 0.20	0.72 ± 0.26	0.841	1.18 ± 0.27
P21–28				
EPSP amplitude (mV)	3.72 ± 0.76	3.23 ± 0.98	0.456	3.43 ± 0.64
EPSP duration (ms)	124.90 ± 21.43	106.08 ± 16.99	0.591	112.72 ± 13.13
EPSP rise time (ms)	4.29 ± 0.92	5.34 ± 1.07	0.660	4.97 ± 0.76
EPSP decay time (ms)	58.91 ± 10.70	48.88 ± 8.14	0.591	52.42 ± 6.39
Short term plasticity (2 *vs*. 1)	0.81 ± 0.11	0.94 ± 0.07	0.333	0.89 ± 0.06
Short term plasticity (3 *vs*. 1)	0.69 ± 0.14	0.72 ± 0.13	0.880	0.71 ± 0.10
Short term plasticity (4 *vs*. 1)	0.48 ± 0.09	0.51 ± 0.10	0.836	0.50 ± 0.07
Short term plasticity (5 *vs*. 1)	0.41 ± 0.06	0.44 ± 0.10	0.872	0.43 ± 0.06
Short term plasticity (6 *vs*. 1)	0.47 ± 0.10	0.46 ± 0.13	0.949	0.46 ± 0.09
EPSC amplitude (pA)	68.94 ± 17.25	70.69 ± 25.93	0.633	69.91 ± 15.88
EPSC duration (ms)	39.31 ± 5.03	36.88 ± 5.39	0.955	38.18 ± 3.56
EPSC rise time (ms)	2.38 ± 0.22	2.32 ± 0.22	0.779	2.35 ± 0.15
EPSC decay time (ms)	17.27 ± 2.55	16.63 ± 2.14	0.867	16.97 ± 1.63
NMDA receptor current (pA)	16.25 ± 3.46	41.34 ± 9.14	0.051	43.26 ± 10.88
AMPA receptor current (pA)	21.80 ± 8.35	61.65 ± 16.37	0.181	29.76 ± 6.15
NMDA/AMPA ratio	1.10 ± 0.23	0.63 ± 0.14	0.101	0.85 ± 0.14
P35+				
EPSP amplitude (mV)	2.79 ± 0.82	3.67 ± 0.33	0.200	2.83 ± 0.42
EPSP duration (ms)	76.18 ± 13.07	76.30 ± 11.04	0.815	77.03 ± 7.65
EPSP rise time (ms)	4.69 ± 0.78	7.06 ± 1.14	0.139	5.50 ± 0.68
EPSP decay time (ms)	35.17 ± 6.46	32.29 ± 4.74	0.815	33.94 ± 3.66
Short term plasticity (2 *vs*. 1)	0.77 ± 0.09	0.96 ± 0.13	0.240	0.92 ± 0.06
Short term plasticity (3 *vs*. 1)	0.51 ± 0.07	0.78 ± 0.08	**0.023**	0.74 ± 0.06
Short term plasticity (4 *vs*. 1)	0.32 ± 0.05	0.59 ± 0.12	**0.044**	0.54 ± 0.06
Short term plasticity (5 *vs*. 1)	0.31 ± 0.04	0.49 ± 0.14	0.240	0.48 ± 0.06
Short term plasticity (6 *vs*. 1)	0.29 ± 0.05	0.55 ± 0.19	0.207	0.49 ± 0.08
EPSC amplitude (pA)	58.74 ± 15.32	70.72 ± 14.97	0.743	56.24 ± 8.21
EPSC duration (ms)	26.83 ± 4.88	24.40 ± 2.72	0.918	26.83 ± 2.43
EPSC rise time (ms)	2.80 ± 0.36	2.97 ± 0.21	0.408	2.86 ± 0.18
EPSC decay time (ms)	9.61 ± 2.46	10.51 ± 2.22	0.758	10.74 ± 1.29
NMDA receptor current (pA)	20.73 ± 6.88	14.35 ± 2.83	0.705	17.69 ± 3.82
AMPA receptor current (pA)	26.32 ± 6.12	24.71 ± 8.71	0.557	25.57 ± 5.11
NMDA/AMPA ratio	0.81 ± 0.15	0.85 ± 0.15	0.973	0.83 ± 0.11

Data are given as means ± SEM, statistical comparisons by Mann–Whitney *U* and *t* tests. *P*‐values shown in bold are statistically significant.

In contrast to the observation for corticostriatal inputs, where the NMDA/AMPA ratio changes occurred largely after P9–12, the major change in NMDA/AMPA ratio of thalamostriatal inputs to D1 and D2 SPNs occurred earlier between the first and the second postnatal week (P3–6: 2.28 ± 0.29 and P9–12: 1.18 ± 0.27; P3–6 *vs*. P9–12: *P* = 0.009, Mann–Whitney *U* test, *n* = 17 and 15; Fig. 5 *D*). This early developmental change in the NMDA/AMPA ratio resulted from a large increase in AMPA/kainate receptor‐mediated currents, whereas the NMDA receptor‐mediated currents did not significantly change (AMPA: P3–6: 5.05 ± 0.80 pA; P9–12: 13.18 ± 2.24 pA; P21–28: 29.76 ± 6.15 pA; and P35+: 25.57 ± 5.11 pA; P3–6 *vs*. P9–12: *P* = 0.001, Mann–Whitney *U* test; and NMDA: P3–6: 11.25 ± 2.12 pA; P9–12: 10.42 ± 2.14 pA; P21–28: 43.26 ± 10.88 pA; and P35+: 17.69 ± 3.82 pA; P3–6 *vs*. P9–12: *P* = 0.271, Mann–Whitney *U* test, *n* = 17, 15, 13 and 21; Fig. 5 *E*). After the second postnatal week, both NMDA and AMPA/kainate receptor‐mediated currents changed concurrently resulting in a constant NMDA/AMPA ratio (P21–28: 0.85 ± 0.14 and P35+: 0.83 ± 0.11, *n* = 13 and 21; Fig. 5 *D* and *E* and Table 3). Interestingly, we observed a drop in both NMDA and AMPA/kainate receptor‐mediated currents in the P35+ range but this did not reach significance.

Similar to the observations of the corticostriatal inputs we found that the thalamostriatal EPSPs also exhibited dynamic changes in EPSP kinetics and exhibited long durations at P9–12 (P3–6: 133.79 ± 13.28 ms; P9–12: 259.13 ± 21.05 ms; P21–28: 112.72 ± 13.13 ms; and P35+: 77.03 ± 7.65 ms; P3–6 *vs*. P9–12: *P* < 0.0001 and P9–12 *vs*. P21–28: *P* < 0.0001; Mann–Whitney *U* test, *n* = 28, 29, 17 and 21; Fig. 5 *F*) as a result of long decay times (P3–6: 69.54 ± 5.87 ms; P9–12: 133.98 ± 9.38 ms; P21–28: 52.42 ± 6.39 ms; and P35+: 33.94 ± 3.66 ms; P3–6 *vs*. P9–12: *P* < 0.0001 and P9–12 *vs*. P21–28: *P* < 0.0001, Mann–Whitney *U* test, *n* = 28, 29, 17 and 21; Fig. 5 *F* and Table 3) and was seen in both D1 and D2 SPNs (Table 3; all *P* > 0.05). The thalamostriatal EPSCs also exhibited the longest duration and decay times at P9–12 (Table 3; EPSC duration: P3–6 *vs*. P9–12: *P* = 0.0044 and EPSC decay time: P3–6 *vs*. P9–12: *P* = 0.0079).

Lastly, we investigated whether presynaptic changes could be observed at thalamostriatal synapses that could affect the short‐term plastic properties of the thalamic synapses onto SPNs. Using trains of electrical stimulation (10 pulses at 20 Hz) we found that thalamic synapses were consistently depressing at all developmental ages but exhibited more pronounced depression on D2 SPNs at P9–12 (*P* = 0.015) and on D1 SPNs at P21–28 (*P* = 0.0001, Fig. 5 *G*).

Combined, these results suggest that the excitatory thalamic synapses onto D1 and D2 SPNs are also functional in the first postnatal week and mostly develop in parallel with the second postnatal week also characterized by long duration EPSPs. The notable exception is a transient stronger thalamic input to D2 SPNs in the second postnatal week. Furthermore we found that, in contrast to the cortical synapses, the thalamostriatal synapses exhibit a rapid change in the NMDA/AMPA ratio as a result of larger increases in AMPA/kainate receptor‐mediated currents from the first to second postnatal week.

### Developmental changes in glutamate receptor expression at striatal excitatory synapses onto D1 and D2 SPNs

We next investigated whether differential expression of certain glutamate receptor types and/or subunit‐containing glutamate receptors (Seeburg, 1993; Monyer *et al*. 1994; Dehorter *et al*. 2011; Lerma & Marques, 2013) might explain our observations of changing EPSP kinetics seen at both corticostriatal (Fig. 4 *F* and Table 2) and thalamostriatal (Fig. 5 *F* and Table 3) synapses onto D1 and D2 SPNs. As both these afferents exhibited similar developmental changes in kinetics, including long duration EPSPs and EPSCs at P9–12, we hypothesized that similar changes might be occurring at both afferents. Therefore, to recruit both excitatory afferents simultaneously, electrical stimulation was performed within the striatum while recording from D1 and D2 SPNs in the presence of GABA receptor antagonists (Fig. 6 *A*). This confirmed the earlier observations of changing EPSP kinetics as they also exhibit both long durations at P9–12 (P3–6: 205.69 ± 23.41 ms; P9–12: 265.73 ± 13.42 ms; and P21–28: 156.68 ± 9.88 ms; P3–6 *vs*. P9–12: *P* = 0.027 and P9–12 *vs*. P21–28: *P* < 0.0001, Mann–Whitney *U* test, *n* = 14, 31 and 24; Fig. 6 *A* and *B*) and decay times (P3–6: 91.60 ± 10.20 ms; P9–12: 126.17 ± 6.59 ms; and P21–28: 40.58 ± 5.59 ms; P3–6 *vs*. P9–12: *P* = 0.015 and P9–12 *vs*. P21–28: *P* < 0.0001, Mann–Whitney *U* test, *n* = 14, 31 and 24; Fig. 6 *A* and *B*).

Next we investigated the contribution of different subunit‐containing NMDA receptors to the evoked EPSP amplitude, duration and decay time using superfusion of the NMDA receptor NR2C/D subunit‐selective antagonist PPDA (200 nm) followed by the addition of the NMDA receptor antagonist d‐AP5 (50 µm) and finally the addition of the AMPA/kainate receptor antagonist NBQX (20 µm). We found that the degree to which these antagonists can block evoked EPSPs exhibited developmental differences. The NR2C/D subunit‐containing NMDA receptors appeared to be highly expressed at P3–6 as superfusion of PPDA (200 nm) maximally reduced the EPSP amplitude (P3–6 *vs*. P21–28; *P* = 0.056, Mann–Whitney *U* test, *n* = 7 and 15), duration (P3–6 *vs*. P21–28; *P* = 0.001, Mann–Whitney *U* test, *n* = 7 and 15) and decay time (P3–6 *vs*. P21–28; *P* = 0.00014, Mann–Whitney *U* test, *n* = 7 and 15; Fig. 6 *C*) at this age. In contrast, the remaining proportion of the NMDA receptor‐mediated current, likely mediated through residual diheteromeric NR2A/B subunit‐containing NMDA receptors (Monyer *et al*. 1994), was most sensitive to superfusion of d‐AP5 (50 µm) at P9–12 in amplitude (P3–6 *vs*. P9–12; *P* = 0.031 and P9–12 *vs*. P21–28; *P* = 0.030, Mann–Whitney *U* test, *n* = 7 and 9 and *n* = 9 and 15, respectively), duration (P3–6 *vs*. P9–12, *P* = 0.029 and P9–12 *vs*. P21–28, *P* = 0.001, Mann–Whitney *U* test, *n* = 7 and 8 and *n* = 8 and 15, respectively) and decay time (P3–6 *vs*. P9–12, *P* = 0.021 and P9–12 *vs*. P21–28, *P* = 0.002, Mann–Whitney *U* test, *n* = 7 and 8 and *n* = 8 and 15, respectively; Fig. 6 *D*). Lastly, application of the AMPA/kainate receptor antagonist NBQX (20 µm) blocked all residual synaptic responses (Fig. 6 *E*), which was greatest at P21–28 on amplitude (P3–6 *vs*. P21–28, *P* = 0.091 and P9–12 *vs*. P21–28, *P* = 0.025, Mann–Whitney *U* test, *n* = 7 and 9 and *n* = 9 and 15, respectively) and in particular on duration (P3–6 *vs*. P21–28, *P* = 0.001 and P9–12 *vs*. P21–28, *P* = 0.002, Mann–Whitney *U* test, *n* = 7 and 8 and *n* = 8 and 15, respectively) and decay time (P3–6 *vs*. P21–28, *P* = 0.001 and P9–12 *vs*. P21–28, *P* = 0.0003, Mann–Whitney *U*, test *n* = 7 and 8 and *n* = 8 and 15, respectively; Fig. 6 *E*). No differences were observed in the effect of these antagonists on synaptic responses between D1 and D2 SPNs (all *P* > 0.05).

Secondly, we investigated the contribution of kainate receptors to the evoked EPSP amplitude, duration and decay time using superfusion of the kainate receptor antagonist UBP‐310 (5 µm), which has broad antagonistic effects on GluK1, GluK2, homomeric GluK3 and GluK5 subunit‐containing kainate receptors (Wisden & Seeburg, 1993; Bahn *et al*. 1994; Bischoff *et al*. 1997; Gallyas *et al*. 2003; Perrais *et al*. 2009; Pinheiro *et al*. 2013). We found a progressive increase across development in the ability of UBP‐310 to block evoked EPSPs, which was maximal at P21–28 (P3–6 *vs*. P21–28, *P* = 0.065, Mann–Whitney *U* test, *n* = 8 and 9; Fig. 6 *F*) suggesting a developmental increase in the expression of kainate receptors. Interestingly, the ability of UBP‐310 to affect the duration and decay time of the evoked EPSP exhibited an inverse relationship, such that at P3–6 and P9–12 superfusion of UBP‐310 resulted in the greatest reduction in duration (P3–6 *vs*. P21–28, *P* = 0.031 and P9–12 and P21–28, *P* = 0.005, Mann–Whitney *U* test, *n* = 7 and 9 and *n* = 14 and 9, respectively) and decay time (P3–6 *vs*. P21–28, *P* = 0.016 and P9–12 *vs*. P21–28, *P* = 0.004, Mann–Whitney *U* test, *n* = 7 and 9 and *n* = 14 and 9, respectively; Fig. 6 *F*), which could suggest further developmental changes in kainate receptor subunit composition. Again, no differences were observed in the response to UBP‐310 between D1 and D2 SPNs (all *P* > 0.05).

In conclusion, we found that glutamate receptor expression at excitatory synapses onto both D1 and D2 SPNs changes across development and can differentially affect the EPSP kinetics. The pharmacological experiments would suggest that in the first postnatal week excitatory synapses contain many NR2C/D subunit‐containing NMDA receptors, followed in the second postnatal week by a transient high expression of the NR2A/B subunit‐containing NMDA receptors. Overall, the expression of AMPA‐ and kainate receptors seems to progressively increase across development.

### Maturation of local inhibitory synaptic connections between striatal SPNs

Our data so far suggests that most of D1 and D2 SPNs can receive excitatory inputs from both the cortex and the thalamus, and are able to generate action potentials allowing them to signal to downstream basal ganglia nuclei during the first postnatal week. However, the probability and timing of these action potentials is under control of inhibition provided by both lateral inhibitory connections between SPNs and inputs from striatal interneurons (Tepper & Plenz, 2006; Ponzi & Wickens, 2010). The analysis of mIPSC frequency would suggest an extended and progressive increase in the number of inhibitory inputs across early postnatal development but whether these arise from neighbouring SPNs or interneurons is unknown. To investigate when SPNs form inhibitory synaptic connections with each other and how these connections change across development, we performed quadruple whole‐cell current‐clamp recordings of SPNs in the first four postnatal weeks (Fig. 7 *A*) including *post hoc* immunocytochemistry (Fig. 7 *B*) and histochemistry (Fig. 7 *C*) to classify recorded neurons as putative D1 or D2 SPNs (see Methods). As immature SPNs have been shown to form gap junctions with each other (Venance *et al*. 2004), with which they can also regulate each other's activity, both hyperpolarizing and depolarizing current steps were used to investigate both gap junctions (Fig. 7 *D*) and synaptic connections (Fig. 7 *E*) between SPNs.

We found that young SPNs were connected through gap junctions during the first postnatal week (P3–6: 3.5%, *n* = 13/377) but progressively lost these connections across development (P9–12: 1.9% and P21–28: 0%, 8/418 and 0/134; P3–6 *vs*. P21–28 *P* = 0.0462, Fisher's exact test; Fig. 7 *F*). This was concurrent with a decrease in coupling coefficient (P3–6: 3.04 ± 0.61 and P9–12: 1.55 ± 0.32, *P* = 0.072, Mann–Whitney *U* test, *n* = 11 and *n* = 10; Table 4) as also reported for other brain regions (Yu *et al*. 2012; Belousov & Fontes, 2013). When the data were split according to SPN type, i.e. connections from D1 to D1, D1 to D2, D2 to D1 and D2 to D2 SPNs, neither gap junction incidence (Fig. 7 *G*) nor other properties of gap junctions (Table 4) were found to differ between the various groups. Combined with the observation that the majority of detected gap junctions were symmetrical (P3–6: 76.9% and P9–12: 75.0%) this would suggest that small groups of young SPNs can form electrically interconnected groups of neurons independent of SPN type.

**Table 4:** Properties of gap junctions between D1 and D2 SPNs

	D1 to D1	D1 to D2	D2 to D1	D2 to D2	All
P3–6					
Amplitude (mV)	1.25 ± 0.18	1.02 ± 0.22	1.19 ± NA	0.86 ± NA	0.92 ± 0.13
Rise time (ms)	116.45 ± 14.65	125.75 ± 2.75	125.00 ± NA	146.50 ± NA	107.02 ± 8.57
Coupling coefficient (%)	1.50 ± 0.07	6.28 ± 1.31	4.40 ± NA	3.03 ± NA	3.04 ± 0.61
Junctional conductance (pS)	194.02 ± 12.96	221.82 ± 52.56	182.88 ± NA	605.25 ± NA	327.90 ± 64.56
P9–12					
Amplitude (mV)	NA ± NA	2.37 ± NA	0.48 ± 0.21	2.16 ± NA	1.11 ± 0.30
Rise time (ms)	NA ± NA	83.60 ± NA	97.10 ± 17.80	85.20 ± NA	106.88 ± 13.66
Coupling coefficient (%)	NA ± NA	3.33 ± NA	1.51 ± 0.71	2.64 ± NA	1.55 ± 0.32
Junctional conductance (pS)	NA ± NA	385.25 ± NA	2612.83 ± 1146.57	411.29 ± NA	1911.85 ± 407.34
P21–28					
Amplitude (mV)	NA ± NA	NA ± NA	NA ± NA	NA ± NA	NA ± NA
Rise time (ms)	NA ± NA	NA ± NA	NA ± NA	NA ± NA	NA ± NA
Coupling coefficient (%)	NA ± NA	NA ± NA	NA ± NA	NA ± NA	NA ± NA
Junctional conductance (pS)	NA ± NA	NA ± NA	NA ± NA	NA ± NA	NA ± NA

Data are given as means ± SEM. NA, not available.

A different picture emerges for the development of the synaptic inhibitory connections. Although we found that already early in development SPNs can form local inhibitory connections with each other, initially with a low incidence, but progressively increasing with age (P3–6: 2.3%; P9–12: 6.9%; and P21–28: 12.2%; *n* = 9/379, 28/408 and 24/197; P3–6 *vs*. P21–28, *P* = 0.0431, Fisher's exact test; Fig. 7 *H*), these connections were not observed equally for all SPN types and they were mostly unidirectional (P3–6: 89%; P9–12: 75%; and P21–28: 68%). Indeed, the earliest synaptic connections at P3–6 were only observed coming from D1 SPNs to D1 and D2 SPNs (D1 to D1: 3.0% and D1 to D2: 3.3%; *n* = 2/67 and *n* = 2/60; Fig. 7 *I*) and no synaptic connections were detected coming from D2 SPN. Only in the second postnatal week between P9 and P12 were synaptic connections observed coming from both D1 and D2 SPNs (Fig. 7 *I*). Interestingly, the relative incidence and observed biases in synaptic connectivity seen in adulthood between D1 and D2 SPNs (Taverna *et al*. 2008; Planert *et al*. 2010) was already apparent at this time including a high incidence of synaptic connections between D2 SPNs (P9–12: D1 to D1: 6.5%; D1 to D2: 5.1%; D2 to D2: 12.8%; and D2 to D1: 5.3%; *n* = 3/46, *n* = 4/78, 13/102 and 4/76; Fig. 7 *I*). These relative biases in synaptic connectivity were even more pronounced by P21–28 (D1 to D1: 7.1%; D1 to D2: 8.0%; D2 to D2: 21.1%; and D2 to D1: 13.9%; *n* = 1/14, *n* = 2/25, 12/57 and 5/36; Fig. 7 *I*) and were consistent with those previously described (Taverna *et al*. 2008; Planert *et al*. 2010). Not only were the D2–D2 SPN connections most numerous at P9–12 and P21–28 (P9–12: 12.8% and P21–28: 21.1%) they also formed the strongest synaptic connections (P9–12: 0.97 ± 0.32 mV and P21–28: 0.72 ± 0.28 mV; Table 5) (Planert *et al*. 2010). Lastly, no significant differences in other properties of the synaptic connections were observed between the different SPN types or across the age ranges (Table 5).

**Table 5:** Properties of unitary GABAergic synapses between D1 and D2 SPNs

	D1 to D1	D1 to D2	D2 to D2	D2 to D1	All
P3–6					
Amplitude (mV)	0.26 ± 0.11	13.7 ± NA	NA ± NA	NA ± NA	2.11 ± 1.46
Duration (ms)	57.00 ± 16.24	42.00 ± NA	NA ± NA	NA ± NA	56.97 ± 8.11
Rise time (ms)	11.33 ± 4.03	9.40 ± NA	NA ± NA	NA ± NA	13.56 ± 2.07
Decay time (ms)	47.13 ± 19.55	74.80 ± NA	NA ± NA	NA ± NA	45.00 ± 8.93
Short term plasticity (2 *vs*. 1)	2.82 ± 0.48	0.61 ± NA	NA ± NA	NA ± NA	1.31 ± 0.35
Short term plasticity (3 *vs*. 1)	2.56 ± 1.36	0.63 ± NA	NA ± NA	NA ± NA	1.02 ± 0.31
Short term plasticity (4 *vs*. 1)	1.26 ± 0.24	0.47 ± NA	NA ± NA	NA ± NA	0.96 ± 0.23
Short term plasticity (5 *vs*. 1)	0.68 ± 0.18	0.52 ± NA	NA ± NA	NA ± NA	0.62 ± 0.10
Short term plasticity (6 *vs*. 1)	1.26 ± 0.41	0.59 ± NA	NA ± NA	NA ± NA	0.81 ± 0.20
P9–12					
Amplitude (mV)	0.10 ± NA	0.68 ± 0.28	0.97 ± 0.32	0.41 ± 0.13	0.95 ± 0.28
Duration (ms)	28.70 ± NA	63.80 ± 13.93	78.62 ± 10.67	111.03 ± 12.98	79.17 ± 7.10
Rise time (ms)	9.4 ± NA	7.83 ± 2.89	9.52 ± 0.96	18.30 ± 1.40	10.37 ± 0.90
Decay time (ms)	10.90 ± NA	42.07 ± 8.31	56.45 ± 7.95	82.67 ± 12.98	56.05 ± 5.62
Short term plasticity (2 *vs*. 1)	1.67 ± NA	1.24 ± 0.71	0.86 ± 0.13	0.61 ± 0.11	0.84 ± 0.12
Short term plasticity (3 *vs*. 1)	1.98 ± NA	2.11 ± 0.97	0.70 ± 0.11	0.44 ± 0.14	0.63 ± 0.10
Short term plasticity (4 *vs*. 1)	3.31 ± NA	1.19 ± 0.49	0.98 ± 0.30	0.36 ± 0.80	0.83 ± 0.15
Short term plasticity (5 *vs*. 1)	1.33 ± NA	1.82 ± 1.08	0.64 ± 0.16	0.47 ± 0.20	0.61 ± 0.11
Short term plasticity (6 *vs*. 1)	0.92 ± NA	1.30 ± 0.58	0.76 ± 0.14	0.31 ± 0.09	0.66 ± 0.12
P21–28					
Amplitude (mV)	0.35 ± NA	0.42 ± 0.08	0.72 ± 0.28	0.54 ± 0.30	0.54 ± 0.13
Duration (ms)	110.00 ± NA	98.85 ± 40.15	50.97 ± 11.45	39.97 ± 5.06	55.29 ± 7.74
Rise time (ms)	11.0 ± NA	10.75 ± 3.65	6.20 ± 1.58	6.18 ± 1.97	6.56 ± 0.99
Decay time (ms)	73.0 ± NA	73.25 ± 27.75	32.28 ± 11.17	14.32 ± 2.06	33.44 ± 6.65
Short term plasticity (2 *vs*. 1)	1.00 ± NA	0.70 ± 0.24	0.42 ± 0.05	0.68 ± 0.12	0.64 ± 0.09
Short term plasticity (3 *vs*. 1)	1.02 ± NA	0.40 ± 0.11	0.55 ± 0.15	0.58 ± 0.16	0.60 ± 0.07
Short term plasticity (4 *vs*. 1)	0.88 ± NA	0.16 ± 0.10	0.45 ± 0.09	0.60 ± 0.18	0.48 ± 0.08
Short term plasticity (5 *vs*. 1)	0.44 ± NA	0.60 ± 0.25	0.37 ± 0.09	0.61 ± 0.17	0.55 ± 0.09
Short term plasticity (6 *vs*. 1)	0.88 ± NA	0.47 ± 0.15	0.52 ± 0.06	0.59 ± 0.18	0.58 ± 0.08

Data are given as means ± SEM.

Together, these results demonstrate that as the striatal circuit matures, symmetric gap junctions between both D1 and D2 SPNs are gradually replaced with precise unidirectional local inhibitory synaptic connections. Moreover, these inhibitory synaptic connections exhibit biases, such as the high incidence of connections between D2 SPNS, and are already established by the second postnatal week.

## Discussion

In this paper we describe the developmental trajectory of identified D1 and D2 SPNs during the first postnatal weeks. We found that the striatal cellular and circuit properties are highly dynamic during this period but several general observations can be made. Firstly, young D1 SPNs are electrically more mature and intrinsic differences in the electrical properties of D1 and D2 SPNs are apparent by the second postnatal week and maintained into adulthood. Secondly, both D1 and D2 SPNs initially exhibit small radially oriented dendrites, which further develop in parallel including increases in length, complexity and spine density. Thirdly, we found that early excitatory synapses onto D1 and D2 SPNs are functional and indeed most SPNs receive long‐range excitatory synaptic inputs from both cortex and thalamus in the first postnatal week. Both inputs progressively strengthen through dynamic changes in postsynaptic glutamate receptor expression, which occurs relatively rapidly for thalamic synapses. Furthermore, we found that excitatory synapses in the second postnatal week exhibit several unique features including a transient strong thalamic drive to D2 SPNs, a stronger NMDA receptor‐mediated cortical input to D1 SPNs, as well as long duration EPSPs. Fourthly, although we found that inhibitory synapses onto D1 and D2 SPNs are functional in the first postnatal week, the development of inhibitory synaptic connections is overall more protracted. Indeed, initially SPNs communicate locally through gap junctions, which are progressively replaced by precise inhibitory synaptic connections in the second and later postnatal weeks. Interestingly, clear biases in inhibitory connections between D1 and D2 SPNs are already apparent in the second postnatal week and are maintained into adulthood. Overall, these findings suggest that early postnatal development of D1 and D2 SPNs follows a dynamic but organized trajectory with many of the cellular and circuit properties established soon after birth.

### Intrinsic cellular properties of D1 and D2 SPNs

We found a progressive development of both the intrinsic electrophysiological and the morphological properties of the D1 and D2 SPNs. Both SPNs are able to generate small ‘immature’ action potentials in the first postnatal days and both undergo a progressive decrease in their input resistance and a hyperpolarization of their resting membrane potential (Lieberman *et al*. 2018), concurrent with an ability to generate large ‘mature’ action potentials at higher firing frequencies (Peixoto *et al*. 2016). However, some differences were observed. Initially, the size and duration of the action potentials were more mature for the D1 SPNs, possibly as a result of their suggested earlier birthdate (Marchand & Lajoie, 1986; van der Kooy & Fishell, 1987; Kelly *et al*. 2018). Secondly, many of the differences in the electrical properties of SPNs, such as the comparatively more depolarized resting membrane potential, higher input resistance and higher firing frequencies of D2 SPNs, are already apparent in the second postnatal week and are maintained into adulthood (Gertler *et al*. 2008; Peixoto *et al*. 2016; Lieberman *et al*. 2018). Morphologically we found that SPNs exhibit a radial dendritic morphology from birth, which undergoes a substantial elaboration concomitant with an increase in dendritic spine density occurring in parallel for the D1 and D2 SPNs. It was not possible to distinguish between D1 and D2 SPNs at any of the age ranges suggesting that previously described differences in morphology (e.g. the increased dendritic arbourization of D1 SPNs (Gertler *et al*. 2008; Benthall *et al*. 2018) or spine density (Gertler *et al*. 2008; Kozorovitskiy *et al*. 2012) might be due to specifics of age, mouse line or methodology (Bagetta *et al*. 2011; Kramer *et al*. 2011; Chan *et al*. 2012; Nelson *et al*. 2012). Overall we found a progressive increase in dendritic spine density consistent with previous studies (Sharpe & Tepper, 1998; Tepper *et al*. 1998), although total spine density is lower compared to two‐photon imaging and serial section EM studies (Ingham *et al*. 1998; Day *et al*. 2006; Kozorovitskiy *et al*. 2012) likely due to our use of DAB immunohistochemistry and occlusion of spines on the top and bottom of dendrites by the DAB reaction product (Ingham *et al*. 1998; Day *et al*. 2006).

### Functional excitatory synaptic inputs onto D1 and D2 SPNs

We found that already soon after birth excitatory inputs onto SPNs are functional and are able to depolarize both D1 and D2 SPNs. Whereas the frequency of mEPSCs in the first postnatal week is close to that observed in adulthood, a large increase in mEPSC amplitude is seen for both D1 and D2 SPNs from the first to second postnatal week, consistent with previous observations (Dehorter *et al*. 2009; Peixoto *et al*. 2016), and suggestive of postsynaptic changes. Indeed, in recordings of electrically evoked corticostriatal and thalamostriatal responses we found that the majority of D1 and D2 SPNs have functional synapses in the first postnatal week and both exhibit similar increases in amplitude (Day *et al*. 2006). This increase in response amplitudes correlates with larger AMPA/kainate receptor‐mediated currents and concomitant decreases in the NMDA/AMPA ratio seen in both D1 and D2 SPNs (Colwell *et al*. 1998; Hurst *et al*. 2001; Peixoto *et al*. 2016). Interestingly, this occurs rapidly at thalamostriatal synapses from the first to second postnatal week whereas the corticostriatal synapses exhibit a more gradual maturation extending into later postnatal weeks. Overall we found that the second postnatal week exhibits many interesting features in that thalamic inputs to D2 SPNs are transiently larger in amplitude as well as D1 SPNs exhibiting larger NMDA receptor‐mediated inputs from cortex, which could result from transient changes in receptor expression or differential maturation of inputs. Furthermore, the EPSP kinetics during this period are characterized by their long durations and decay times. Pharmacological study of glutamate receptor expression at these excitatory synapses would suggest that their expression is highly dynamic in these early postnatal weeks with a progressive decrease in NR2C/D subunit‐containing NMDA receptors, consistent with previous observations (Monyer *et al*. 1994; Dehorter *et al*. 2011), a transient and significant increase in the expression of NR2A/B subunit‐containing NMDA receptors in the second postnatal week and a gradual increase in AMPA/kainate receptor expression. The expression of UBP‐310‐sensitive kainate receptors seems to also progressively increase, whereas their contribution to the EPSP duration and decay time kinetics at later stages of development seems to decrease, suggesting complex developmental changes in glutamate receptor expression (Wisden & Seeburg, 1993; Bahn *et al*. 1994; Bischoff *et al*. 1997). The different subunits that make up the NMDA receptors and kainate receptors affect their channel kinetics (Monyer *et al*. 1994; Flint *et al*. 1997; Cull‐Candy & Leszkiewicz, 2004; Chen *et al*. 2006; Lerma & Marques, 2013) and, in combination with changes in the membrane time constant (Spruston *et al*. 1994), might well contribute to the long duration EPSPs seen in the second postnatal week. Indeed, the observation of similar developmental changes in EPSC duration and decay time suggest that the observed effects cannot be explained solely by developmental changes in the membrane time constant. These long duration EPSPs could well play a role in facilitating synaptic integration and synaptic plasticity (Carmignoto & Vicini, 1992; Tang *et al*. 1999; Frerking & Ohliger‐Frerking, 2002; Fino *et al*. 2010) at excitatory synapses on SPNs during this period and the generation of ensemble activity (Carrillo‐Reid *et al*. 2008). Finally, the observation of a relatively stable mEPSC frequency was surprising in light of reported increases in the density of asymmetric glutamatergic synapses during this period (Butler *et al*. 1998; Sharpe & Tepper, 1998; Tepper *et al*. 1998) and observed increases in dendritic spine density and previous reported increases in EPSC frequency (Peixoto *et al*. 2016). Possible explanations for our observations but also others (Dehorter *et al*. 2011) could include biases towards recording mEPSCs mediated by axo‐dendritic synapses instead of axo‐spinous synapses, as the density of axo‐dendritic synapses have been shown to remain stable during development (Sharpe & Tepper, 1998), or could result from a dynamic interplay between synapse number and release probability in which increases in synapse number are balanced by decreases in release probability (Choi & Lovinger, 1997).

### Local inhibitory synaptic connections between D1 and D2 SPNs

The activity of striatal SPNs and their response to excitatory input is modulated by inhibition coming from local collaterals from neighbouring SPNs (Somogyi *et al*. 1981; Bolam & Izzo, 1988; Taverna *et al*. 2008; Planert *et al*. 2010; Cepeda *et al*. 2013) and striatal interneurons (Tepper & Plenz, 2006; Ponzi & Wickens, 2010). Measurements of mIPSCs reveal that both D1 and D2 SPNs already receive some inhibitory input in the first postnatal week. The initial frequency of these events is lower than that seen for mEPSCs and progressively increases in the second and later postnatal weeks as previously observed (Dehorter *et al*. 2011). This would suggest that GABAergic synapse density increases well after the second postnatal week. We also observed a dramatic increase in mIPSC amplitude from the first to second postnatal week consistent with a rapid maturation as a result of an increased number (Nusser *et al*. 1997) and/or changed subunit composition of postsynaptic GABA receptors (Farrant & Nusser, 2005; Arama *et al*. 2015). To investigate when and how SPNs start communicating with each other in the striatum and whether the number of synaptic connections does increase we performed simultaneous quadruple whole‐cell patch‐clamp recordings of SPNs. We found that both D1 and D2 SPNs are mainly connected through gap junctions in the first postnatal week, but both the incidence of gap junctions and their coupling coefficient rapidly decrease and no gap junctions were observed in adulthood, consistent with previous electrophysiological (Venance *et al*. 2004; Yu *et al*. 2012) and dye coupling experiments (Tepper *et al*. 1998). These initial gap junctions could facilitate synchronization of SPN activity (Venance *et al*. 2004; Hestrin & Galarreta, 2005) and the establishment of synaptic connections (Yu *et al*. 2012). The first inhibitory synaptic connections were detected in the first postnatal week coming from D1 SPNs only, potentially a reflection of their earlier birthdate (Marchand & Lajoie, 1986; van der Kooy & Fishell, 1987; Kelly *et al*. 2018), but in the second postnatal week both D1 and D2 SPNs form inhibitory synaptic connections with each other. Interestingly, the relative biases in connectivity (e.g. the high interconnectivity between D2 SPNs) seen in adulthood in this study and by others (Taverna *et al*. 2008; Planert *et al*. 2010) are already established by the second postnatal week raising the question of what instructs SPNs to form these precise intrastriatal circuit motifs (Plenz, 2003).

In conclusion, we show that early postnatal development of the intrinsic cellular and circuit properties of the D1‐expressing direct pathway and the D2‐expressing indirect pathway SPNs is highly dynamic but follows a clear developmental trajectory. Moreover, we show that many of the properties of mature D1 and D2 SPNs are already apparent by the first and second postnatal weeks, which is thought to be a period prior to much exploratory motor behaviour (Dehorter *et al*. 2011) or exposure to structured input from the sensory periphery (Tobach *et al*. 1971; Krug *et al*. 2001; Akerman *et al*. 2002; Ko *et al*. 2013; Mowery *et al*. 2015, 2016, 2017). This is consistent with the idea that neuronal specification can occur early in development (Lobo *et al*. 2006, 2008; Arlotta *et al*. 2008; Ehrman *et al*. 2013; Lu *et al*. 2014; Zhang *et al*. 2016; Merchan‐Sala *et al*. 2017; Kelly *et al*. 2018; Tinterri *et al*. 2018; Xu *et al*. 2018) with further postnatal development guided by, for example, neural activity (Zhang & Poo, 2001; Kozorovitskiy *et al*. 2012; Peixoto *et al*. 2016) and neuromodulation (Kozorovitskiy *et al*. 2015; Lieberman *et al*. 2018). Future work will be able to clarify the precise interaction of these factors during striatal development as well as their differential involvement in neurodevelopmental disorders (Graybiel & Rauch, 2000; Del Campo *et al*. 2011; Langen *et al*. 2011; McNaught & Mink, 2011; Shepherd, 2013; Albin, 2018).

## Additional information

### Competing interests

We declare no conflict of interest.

### Author contributions

R.K., F.v.H., F.E. and T.J.E. performed the electrophysiology experiments and analysis. F.v.H. and A.M.D. performed anatomical experiments and analysis. All authors discussed the data. T.J.E. wrote the manuscript. All authors have read and approved the final version of this manuscript and agree to be accountable for all aspects of the work in ensuring that questions related to the accuracy or integrity of any part of the work are appropriately investigated and resolved. All persons designated as authors qualify for authorship, and all those who qualify for authorship are listed.

### Funding

T.J.E. was supported by an MRC Career Development Award (MR/M009599/1) and AMD by an Imperial College research bursary.
